# *KLHL21*, a novel gene that contributes to the progression of hepatocellular carcinoma

**DOI:** 10.1186/s12885-016-2851-7

**Published:** 2016-10-21

**Authors:** Lei Shi, Wenfa Zhang, Fagui Zou, Lihua Mei, Gang Wu, Yong Teng

**Affiliations:** 1 School of Life Sciences, Chongqing University, Chongqing, 400044 People’s Republic of China; 2 Third Affiliated Hospital, Third Military Medical University, Chongqing, 400044 People’s Republic of China; 3 Department of Oral Biology, Dental College of Georgia, Augusta University, Augusta, GA 30912 USA; 4 GRU Cancer Center, Medical College of Georgia, Augusta University, Augusta, GA 30912 USA; 5 Department of Biochemistry and Molecular Biology, Medical College of Georgia, Augusta University, Augusta, GA 30912 USA

**Keywords:** *KLHL21*, Bioinformatics, HCC, Biomarker

## Abstract

**Background:**

Hepatocellular carcinoma (HCC) has very high prevalence and associated-mortality. However, targeted therapies that are currently used in clinical practice for HCC have certain limitations, in part because of the lack of reliable and clinically applicable biomarkers that can be used for diagnosis and prognosis assessments and for the surveillance of treatment effectiveness.

**Methods:**

Meta-analysis was used to analyze the integrated microarray data for global identification of a set of robust biomarkers for HCC. Quantitative RT-PCR (qRT-PCR) was performed to validate the expression levels of selected genes. Gene expression was inhibited by siRNA. CellTiter 96^®^ AQueous One Solution Cell Proliferation assays were used to determine cell proliferation, and Transwell assays were used to determine cell migration and invasion potential.

**Results:**

Meta-analysis of the expression data provided a gene expression signature from a total of 1525 patients with HCC, showing 1529 up-regulated genes and 478 down-regulated genes in cancer samples. The expression levels of genes having strong clinical significance were validated by qRT-PCR using primary HCC tissues and the paired adjacent noncancerous liver tissues. Up-regulation of *VPS45*, *WIPI1*, *TTC1*, *IGBP1* and *KLHL21* genes and down-regulation of *FCGRT* gene were confirmed in clinical HCC samples. *KLHL21* was the most promising gene for potential use as a bioclinical marker in this analysis. Abrogating expression of it significantly inhibited cell proliferation, migration and invasion.

**Conclusions:**

Our study suggests that *KLHL21* is a potential target for therapeutic intervention. Our findings also provide novel candidate genes on a genome-wide scale, which may have significant impact on the design and execution of effective therapy of HCC patients.

**Electronic supplementary material:**

The online version of this article (doi:10.1186/s12885-016-2851-7) contains supplementary material, which is available to authorized users.

## Background

Hepatocellular carcinoma (HCC) is the most common primary malignancy of the liver and the second leading cause of cancer death in men worldwide [1]. In patients with HCC, the prediction of prognosis is more complex compared with other solid tumors since there is no worldwide consensus on the use of any HCC staging system [2, 3]. Clinical studies demonstrate that only one-third of the newly diagnosed patients are presently eligible for curative treatments [4] and the 5-year survival after resection for early-stage HCC ranges from 17 to 53 % with recurrence rate as high as 70 % [5]. Therefore, prognosis estimation and indicators for successful treatment options are critical steps in the management of patients with HCC.

Genes that are commonly dysregulated in cancer are clinically attractive as candidate prognostic markers and therapeutic targets. Previous bioinformatics analyses of gene expression profiles have revealed targets for predicting prognosis and survival in patients with HCC are involved in angiogenesis, cell cycle regulation, invasion and metastasis [6–11]. Although high-throughput genomic technologies have facilitated the identification of cancer biomarkers and improved our understanding of the molecular basis of tumor progression, the most common drawbacks of these studies are a lack of agreement due to the differences across experimental platforms, sample size and quality, inconsistent annotation, ongoing discovery as well as the methods used for data processing and analysis. Moreover, the number of prognostically-informative genes in HCC varies from 3 to 628, with low predictive accuracy, which leads to inherent difficulties in drawing definitive conclusions [12–15]. Therefore, identification of robust biomarker candidates for HCC provides a novel potential link between clinical prognosis and cancer survival rates.

In this study, a meta-analysis was used to obtain a consistent gene expression signature for HCC using the integrating microarray data. The dysregulated genes with potentially high clinical significance were validated by qRT-PCR, among which *KLHL21* was the most promising. Suppressing its expression inhibited cell proliferation, migration and invasion in HCC cells. Our analyses identified a novel set of HCC biomarkers with high accuracy, using a combination of molecular techniques and clinical information from patients with HCC. This may lead to potential prognostic and therapeutic applications in the future.

## Methods

### Data acquisition, inclusion criteria and study strategy

We searched the published microarray datasets from Gene Expression Omnibus (GEO, http://www.ncbi.nlm.nih.gov/geo/) [16] and ArrayExpress (http://www.ebi.ac.uk/arrayexpress/) [17] up to June 2015, with keyword “hepatocellular carcinoma OR HCC” filtered by organism “Homo sapiens”. To identify new prognostic biomarkers in HCC, the selected microarray datasets must meet the following criteria: (i) both tumor tissues and their adjacent tissues (or normal tissues) were included; (ii) contained contain a large number of patient samples (>50) and high gene coverage (>10,000 filtered genes). After background correction and normalization of raw data, multiple probe sets were reduced to one per-gene symbol using the most variable probe measured by interquartile range (IQR) values across arrays. Significance analysis of microarray (SAM) [18] was used to determine the differentially expressed genes (DEGs), with a false discovery rate (FDR) <0.001 and 1,000 times permutations.

### Functional analysis of DEGs

To investigate the cellular component (CC), molecular function (MF) and biological process (BP) of DEGs, Gene Oncology (GO) enrichment analyses were performed by Database for Annotation, Visualization and Integrated Discovery (DAVID) [19, 20] and WEB-based GEne SeT AnaLysis Toolkit (WebGestalt). To investigate regulatory network, pathway enrichment analyses were performed by BRB-ArrayTools based on KEGG (http://www.genome.jp/kegg/) and BioCarta (http://www.biocarta.com/). In this study, the LS/KS permutation test was used for pathway enrichment and gene-sets with *p* < 0.00001 were considered significant. Co-expression analysis of the DEGs was performed with a Spearman correlation coefficient absolute value > 0.75 (*p* < 10e-10) by Cytoscape [21].

### Survival analysis

To analyze the correlation between gene expression and clinical relevance, the association between the gene expression levels and survival of patients with HCC was analyzed using the GSE10186 entry. In univariate survival analyses, Cox proportional hazard regression model (Wald test) were used to identify factors important for survival followed by 1,000 times permutation test. In univariate survival analyses, Kaplan-Meier method and the log-rank test were used to compare overall survival curves between high and low gene expression groups. For all statistical analyses, *p* < 0.05 were considered significant.

### Literature confirmation

The DEGs identified from meta-analysis were validated by publications and scientific literature available on PubMed (http://www.ncbi.nlm.nih.gov/pubmed/?term=). Keyword used, take gene “MYCN” for example, was “(((((survival[Title/Abstract]) OR prognosis[Title/Abstract]) OR biomarker[Title/Abstract]) AND tumor[Title/Abstract]) OR cancer[Title/Abstract]) AND MYCN[Title/Abstract]”.

### Cell culture and primary tissues

MHCC97H and HCC-LM3 cells were purchased from the Cell Bank of Type Culture Collection of Chinese Academy of Sciences (Shanghai, China) and maintained according to the supplier’s instructions. Twenty-eight primary HCC tissue samples with paired adjacent normal liver tissue samples were collected and all experimental procedures were approved by the IRB of Third Affiliated Hospital of Third Military Medical University (Chongqing, China). None of the patients had received chemotherapy or radiotherapy before or after surgery. Written informed consent was obtained from all patients or their guardians and all samples were histologically confirmed before analysis.

### QRT-PCR analysis

To prepare cDNA, 1 μg total RNA was extracted from cell lines and tissue samples using QIAGEN OneStep RT-PCR Kit. Amplifications of cDNA stocks were performed by qRT-PCR in triplicate using GoTaq qPCR Master Mix (Promega) as described previously [22–24]. In this study, unique primer pairs (Additional file 1: Table S1) used to amplify the selected genes were designed using Primer-Blast at NCBI (http://www.ncbi.nlm.nih.gov/tools/primer-blast/index.cgi) and assessed for secondary structure using M-Fold (http://mfold.rna.albany.edu/). Where possible, the primers were designed to span or include an intron to avoid amplification of genomic DNA and to have similar melting temperatures in the range 56–62 °C. Relative gene expression levels were analyzed by the ΔΔCT method and normalized against *β-actin*.

### Gene silencing by RNA interference

HCC cells were transiently transfected with small interfering RNA (siRNA) using DharmaFECT (Dharmacon, Lafayette, CO). Twenty-one base pair siRNA duplexes targeting *KLHL21* gene (siKLHL21-1: 5′-GTACAACTCAAGCGTGAAT-3′; siKLHL21-2: 5′-TGTCATTGCTGTCGGGTTA-3′) and a standard control (Dharmacon siCONTROL nontargeting siRNA) were synthesized by Dharmacon.

### Cell proliferation, migration and invasion assays

For cell proliferation assays, HCC cells were seeded into 96-well plate at a density of 1 × 10^3^ cells. The cell proliferation rate was analyzed at different time points (1–5 days) with CellTiter 96® AQueous One Solution Cell Proliferation assay (Promega, Madison, WI) according to manufacturer’s instruction. The absorbance at 490 nm was measured with a microplate reader and the average absorbance values from six wells per group were calculated. Quantitative cell migration and invasion assays were performed using 24-well Boyden chambers (Coring, NY, USA) as described previously [22–24]. The numbers of migrated and invaded cells in six randomly selected fields from triplicate chambers were counted in each experiment under a Leica inverted microscope (Deerfield, IL, USA).

### Statistical analysis

Differences in quantitative data between two groups were analyzed using 2-sided paired or unpaired Student t-tests. All of the analyses were performed using SPSS software version 18.0 (SPSS, Chicago, IL, USA). *P* < 0.05 was considered to be statistically significant.

## Results

### The most DEGs in HCC are identified by integrated bioinformatics analysis

**Fig. 1:**
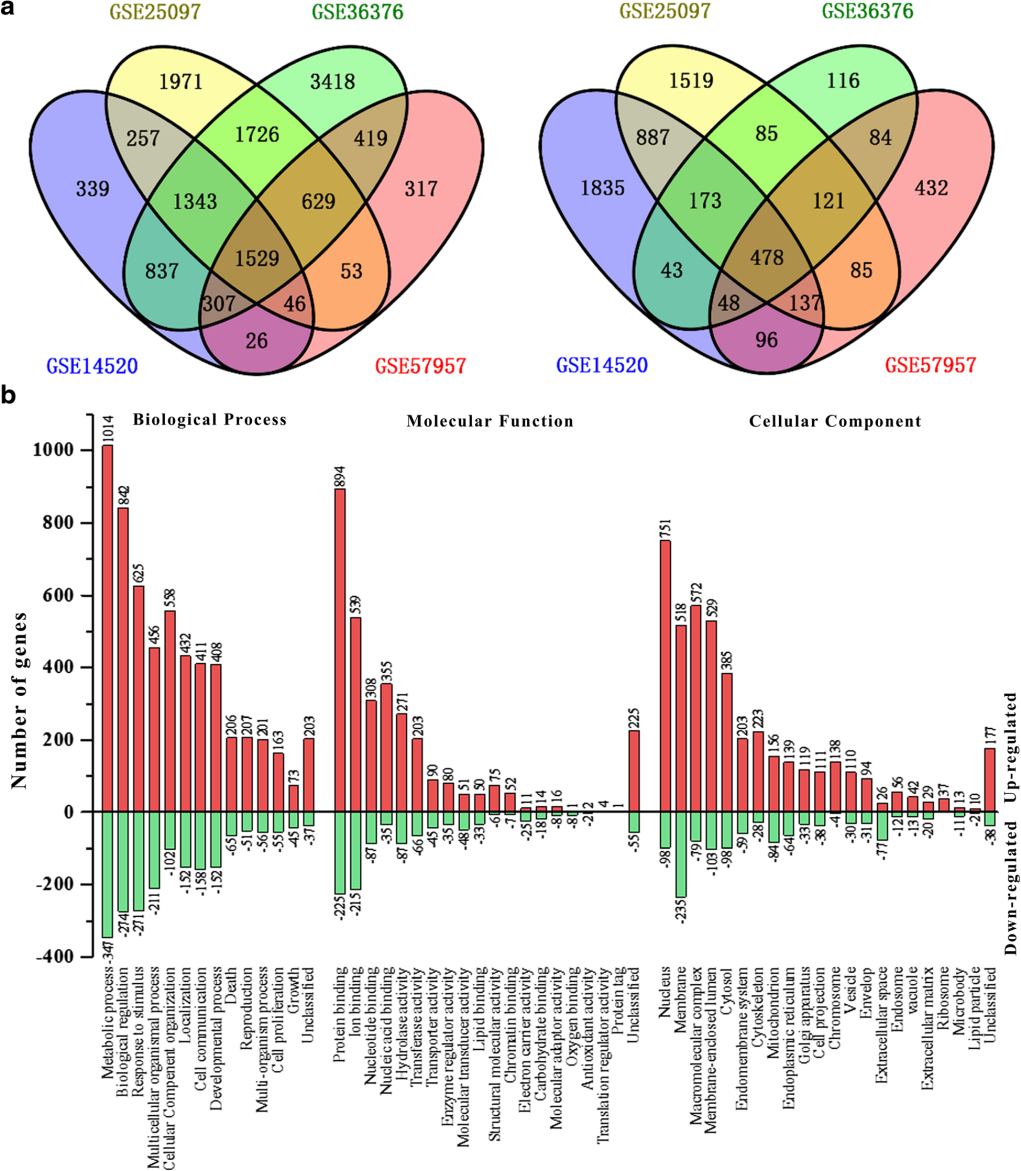
The DEGs in hepatocellular carcinoma are identified by integrated bioinformatics analysis. **a** Venn diagram of up-regulated genes (l*eft*) and down-regulated genes (*right*). **b** GO enrichment analysis was performed to identify enriched BP, CC and MF in both up-regulated genes and down-regulated genes

According to the inclusion criteria (Additional file 2: Figure S1), 4 independent studies (GSE14520, GSE25097, GSE36376 and GSE57957) retrieved from public databases (GEO and ArrayExpress) were used to identify the DEGs in HCC (Additional file 3: Table S2). The technical framework used in the meta-analysis is shown in Additional file 4: Figure S2. After normalization and annotation, SAM was performed to analyze the DEGs from each dataset. Only the DEGs displaying the same trend (*p* < 6.25e-6) in 4 datasets were selected for further analysis. In total, 1529 significantly up-regulated genes and 478 significantly down-regulated genes were identified in HCC samples (Fig. 1a and Additional file 5: Table S3). Hierarchical clustering analyses of these DEGs were depicted using GSE36376 since it had the highest gene coverage and largest samples. Almost completely separate clustering was observed between HCC and noncancerous samples, indicating that the up-regulated and down-regulated genes are differentially expressed in HCC and noncancerous tissues (Additional file 6: Figure S3).

**Fig. 2:**
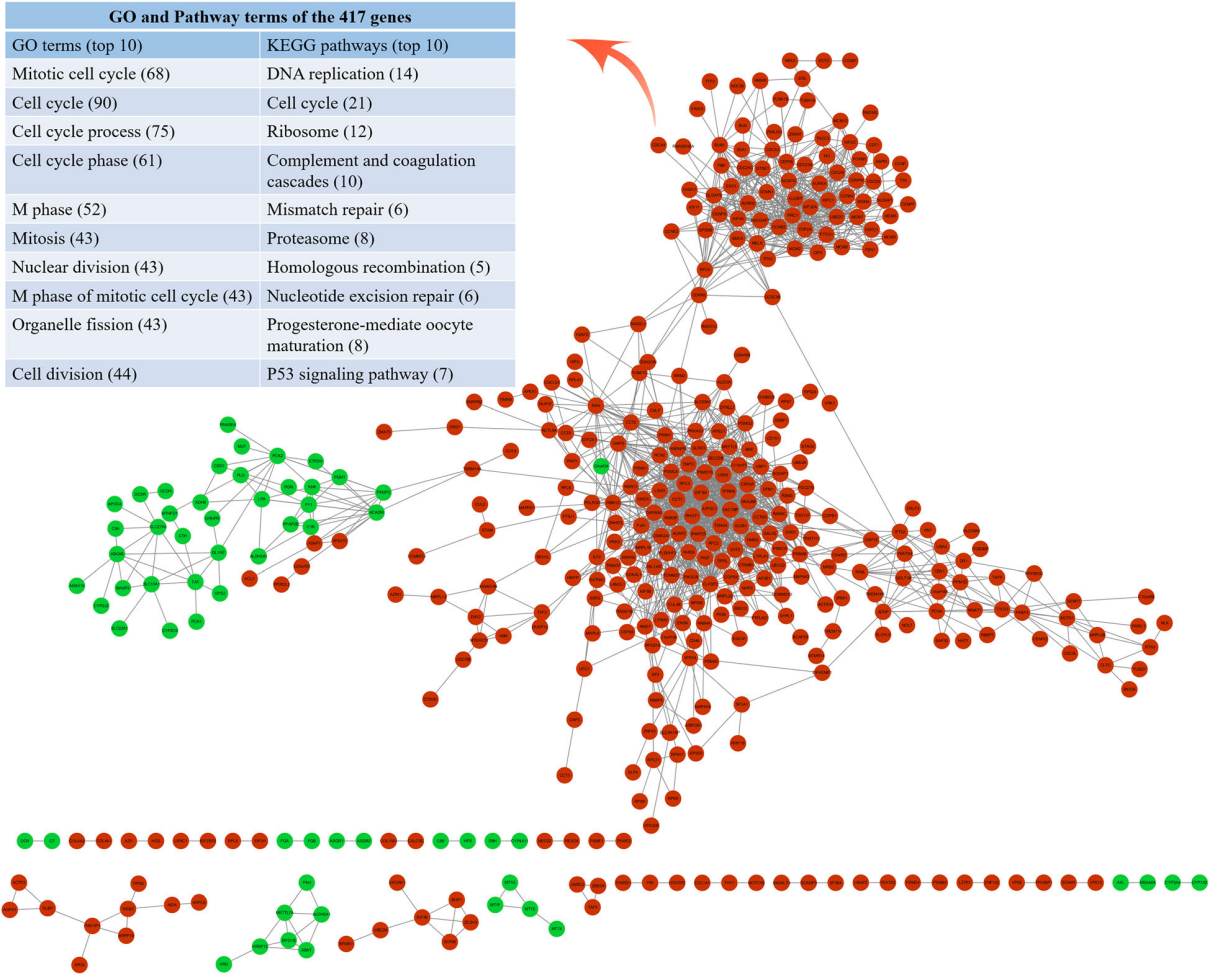
The regulatory network of the DEGs is identified by co-expression, GO and pathway analysis. Each node corresponds to a gene, and a pair of nodes is connected with an edge if there is a significant co-expression relationship between them. *Red*: up-regulated genes; *Green*: down-regulated genes

GO enrichment analyses were used to determine the common functional roles of the DEGs (Fig. 1b). The top three highly enriched GO categories for BP were metabolic process (67.81 %), biological regulation (55.61 %) and response to stimulus (44.64 %), indicating significant changes of cellular metabolism in HCC tissues compared with that in the adjacent tissues. To visualize the interaction of enriched GO, directed acyclic graphs were constructed by the DEGs (Additional file 7: Figure S4 and Additional file 8: Figure S5), showing the main function of the enriched genes was associated with cellular process, metabolic process, and catalytic activity. Furthermore, KEGG and Biocarta analyses were used to investigate the networks of the DEGs. KEGG pathway mapping showed 105 significant pathways for up-regulated genes and 16 significant pathways for down-regulated genes (*p* < 0.00001) in HCC. Gene-sets such as “cell cycle”, “Wnt signaling pathway”, “mTOR signaling pathway” and cancer pathways such as “pathways in cancer” are all significant for the up-regulated genes. Interestingly, 40 genes were enriched in cell cycle pathway (LS/KS permutation test *p* < 0.00001, Additional file 9: Figure S6), suggesting this signaling plays an essential part in HCC development and progression. Using Biocarta enrichment analysis, we identified 62 significant pathways for up-regulated genes and 3 for down-regulated genes. The cell growth pathways, such as “cell cycle: G1/S check point”, “cell cycle: G2/M check point”, “growth hormone signaling pathway”, “signaling of hepatocyte growth factor receptor”, “Ras signaling pathway” and “Wnt signaling pathway”, were also enriched in up-regulated genes. To integrate multiple layers of information and gain new biological insights into the regulatory network of the DEGs, co-expression networks analysis was performed. In this assay, 417 genes were identified to be co-expressed, which were selected as hub genes for GO and KEGG pathway analyses (Fig. 2). Consistently, cell cycle genes were identified by the first 8 significant GO terms as well as KEGG pathways.

### Survival analysis indicates clinical significance of the integrated-signature genes

**Fig. 3:**
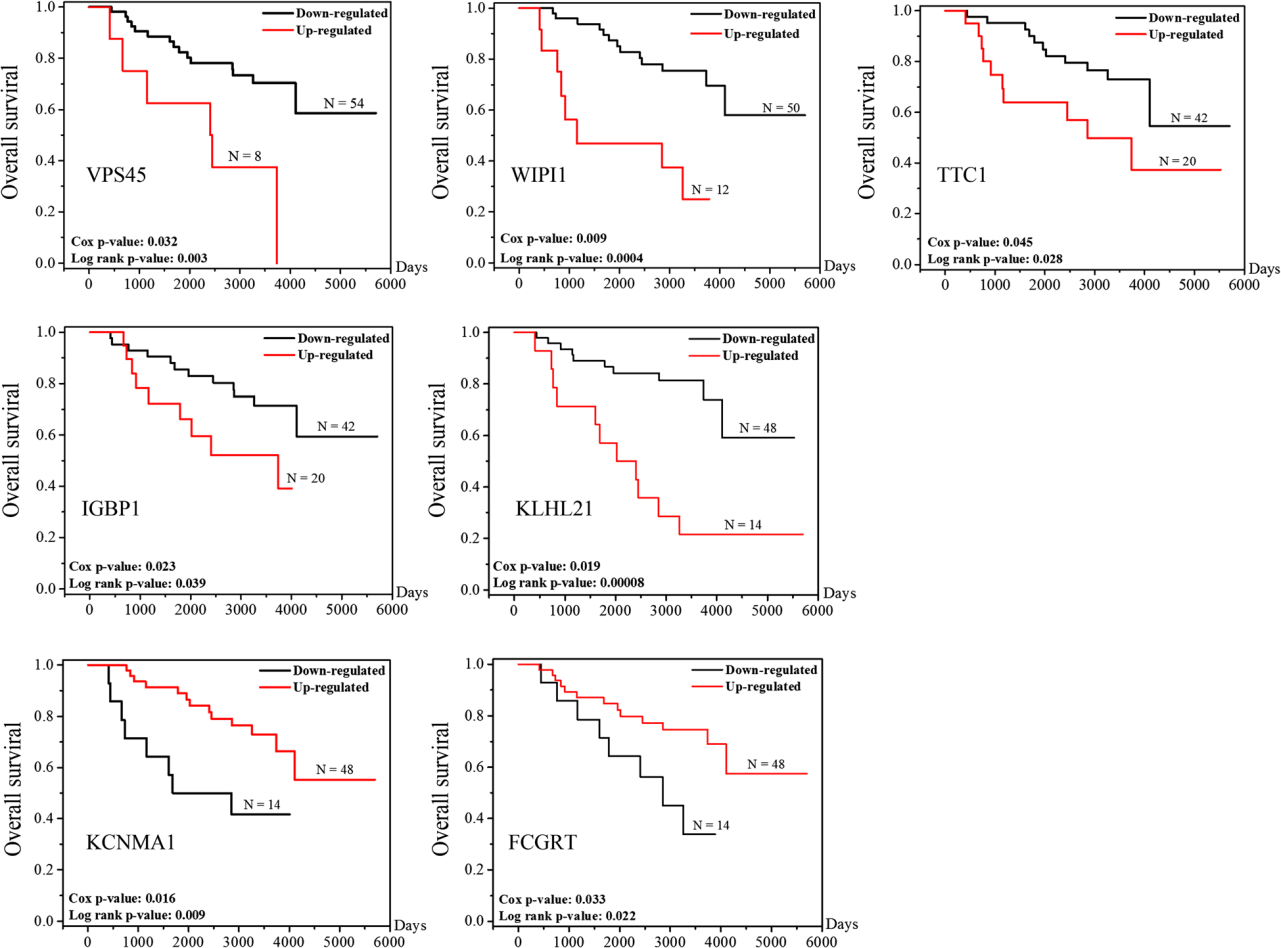
The Kaplan-Meier survival curves (Univariate survival method) for HCC patients with high (in *red*) or low (in *black*) individual gene expression show genes associated with patient survival

To relate the gene expression levels to clinical outcome, survival analysis was performed using the GSE10186 entry. Fifty-nine up-regulated genes and twenty down-regulated genes were associated with overall survival of patients with HCC (Cox *p* < 0.05) (Additional file 10: Table S4). More than 40 % of the DEGs (32 out of 79 genes) have been proven to have prognostic values with at least one type of cancer, including well-known oncogenes *RHEB* and *MYCN* [25–28]. Moreover, ~25 % of other DEGs (20 out of 79 genes) contribute to cell growth/proliferation, invasion/migration, apoptosis/autophagy and differentiation. In further study, 9 up-regulated genes (*VPS45*, *WIPI1*, *SLC9A3R1*, *TTC1*, *GNB5*, *IGBP1*, *MAP3K7*, *KLHL21* and *NOX4*) with a hazard ratio (HR) > 1 and 3 down-regulated genes (*KCNMA1*, *IQGAP2* and *FCGRT*) with a HR < 1 were selected for validation. Among them, *NOX4*, *MAP3K7*, *SLC9A3R1* and *IQGAP2* were well studied in HCC and their expression levels strongly associate with prognostic features [29–34]. Kaplan-Meier survival curve showed for the first time that high expression levels of *VPS45*, *WIPI1*, *TTC1*, *GNB5*, *IGBP1* or *KLHL21* gene or low levels of *KCNMA1* or *FCGRT* gene were significantly correlated with low overall survival of HCC patients (Fig. 3).

### QRT-PCR analysis validates the expression levels of the identified HCC biomarkers in clinical samples

**Fig. 4:**
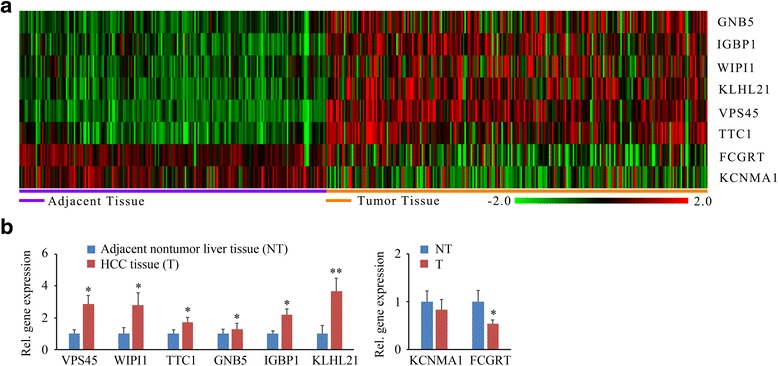
QRT-PCR analysis validates the expression levels of the identified HCC biomarkers in clinical samples. **a** Gene expression profile of the 8 novel genes in adjacent tissues and HCC tissues in GSE36376. **b** qRT-PCR validation of the gene expression in primary HCC tissues and paired adjacent noncancerous liver tissues. Fold change was calculated for the selected genes in HCC tissues relative to paired adjacent normal liver tissues. Error bars represent SD (*n* = 3), *P < 0.05, **P < 0.01

To validate 8 new candidate genes from the above analyses (Fig. 4a), we determined their expression levels from 28 pairs of fresh HCC and adjacent noncancerous liver tissues using qRT-PCR. As shown in Fig. 4b, the average expression levels of *VPS45*, *WIPI1*, *TTC1*, *IGBP1* and *KLHL21* genes in all tested HCC tissues were greatly increased compared with those in the adjacent non-tumor tissues, showing the similar results to microarray data. *FCGRT* was shown to be down-regulated in Meta-analysis (Fig. 4a), and its expression levels were also decreased in primary HCC tissue samples in qRT-PCR assays (Fig. 4b). No significant changes in the expression levels of *GNB5* and *KCNMA1* were observed between HCC tissues and the paired non-tumor tissues (Fig. 4b), which was not consistent with meta-analysis.

### Knockdown of KLHL21 suppresses HCC cell proliferation, migration and invasion

**Fig. 5:**
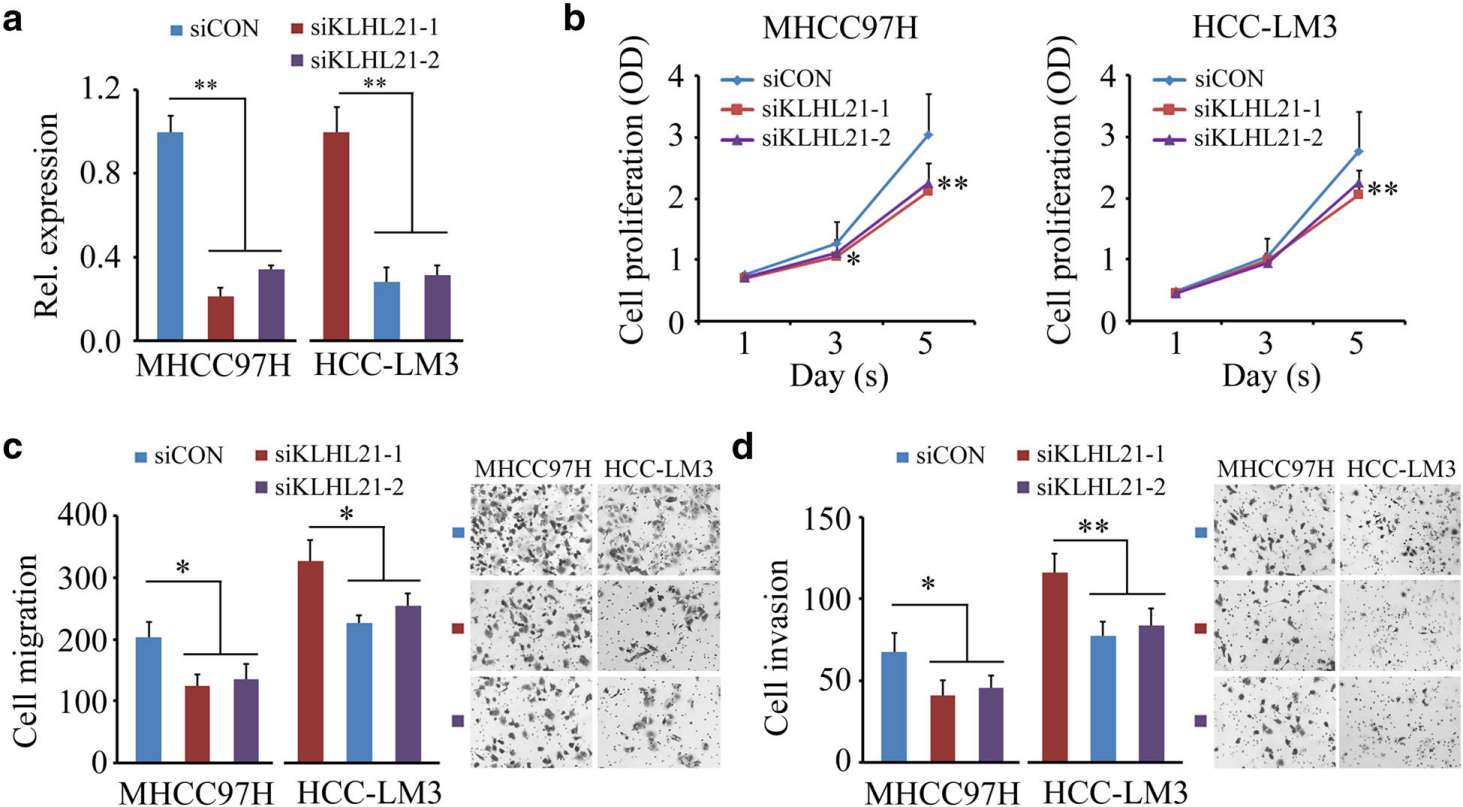
Knockdown of *KLHL21* suppresses HCC cell proliferation, migration and invasion. **a** Effect of siRNA-mediated knockdown of *KLHL2*1 on MHCC97H and HCC-LM3 cells. **b** Effect of *KLHL21* knockdown on cell proliferation. **c** and **d** Effect of *KLHL21* knockdown on cell migration and invasion. Error bars represent SD (*n* = 3), *P < 0.05, **P < 0.01

The expression levels of *KLHL21* were increased most significantly in primary HCC tissues compared with the other validated genes (Fig. 4b). To elucidate the role of *KLHL21* in the progression of HCC, we studied the effects of siRNA-mediated *KLHL21* knockdown on HCC cell proliferation. MHCC97H and HCC-LM3 cell lines have high metastatic potential, and loss of *KLHL21* expression (Fig. 5a) inhibited cell proliferation within 5 days in these cells (Fig. 5b). We next investigated whether *KLHL21* affected cell migration and invasion within 24 h. Transwell assays were carried out to quantitatively determine the effect of *KLHL21* on cell migration. As shown in Fig. 5c, a significantly lower number of *KLHL21* knockdown cells migrated to the lower face of the Transwell membrane compared with that of the knockdown control cells (~40 % reduction in MHCC97H cells and ~30 % reduction in HCC-LM3 cells, respectively). Depletion of *KLHL21* also reduced cell invasion (Fig. 5d). These data suggest that *KLHL21* is critically important for hepatocellular development and progression. Suppression of its expression may provide a novel strategy to efficiently combat HCC.

## Discussion

Meta-analysis has been widely used as a powerful method in searching DEGs in various types of cancers [35–39]. In this study, we systematically identify a set of molecular prognostic markers for HCC using meta-analysis. To minimize the limitation from a single microarray dataset, we examined the overlap among many studies using different platforms in an unbiased manner. By comparing gene expression data from 1525 paired samples profiled in the GEO datasets, and by combining molecular and clinical data to reduce false-positive errors, we demonstrate a core gene set with prognostic potential.

Cancer biomarkers are the measurable molecular changes to either cancerous or normal tissues of patients [40–42]. A reliable biomarker can be used for cancer diagnosis, risk and prognosis assessments, and more importantly, some of them can be exploited as therapeutic targets. Therefore, better understanding of the biological significance of such markers and validation of their usefulness are pivotal for developing novel targeted therapies. HCC appears to be characterized by increased glycolysis, attenuated mitochondrial oxidation, and increased arachidonic acid synthesis [43], suggesting abnormal metabolism in HCC development and progression. In this study, GO analysis, KEGG and BioCarta pathway analyses were performed to determine the roles and pathways of DEGs. These analyses implicate that the expression profiling of metabolism genes was significantly changed in HCC. The deregulated energy metabolism of cancer cells modifies the metabolic pathways and influences various biological processes including cell proliferation. Not surprisingly, the dysregulated genes identified in our study were highly associated with cell cycle pathways.

In order to determine the clinical relevance of the DEGs, survival analysis was performed and 79 DEGs were found to be associated with overall survival. Most of these genes (65.82 %) have prognostic features and strong associations with some cancers. For example, *MYCN* is well-studied biomarker for neuroblastoma and inactivation of it results in impaired cell growth and enhanced cell death in neuroblastoma [44–46]. *RHEB* acts as a proto-oncogene in the appropriate genetic milieu and signaling context, and its overexpression cooperates with *PTEN* haploinsufficiency to promote prostate tumorigenesis [47]. The elevated expression levels of these two genes are also found in our study, suggesting that cancers from different tissues may share common features and these genes can be utilized as pan-cancer biomarkers. The expression levels of *GPC3* are down-regulated to facilitate cell migration, invasion and tumorigenicity in ovarian cancer [48, 49]. However, our study shows that *GPC3* is an up-regulated gene in HCC, which agrees with other studies [50–53]. These observations indicate that the same gene might exhibit opposite effects on different cancer types, and the genes like *GPC3* cannot be used as pan-cancer biomarkers.

The HR derived from the Cox proportional hazards model provides a statistical test of treatment efficacy and an estimate of relative risk of events. Therefore, understanding of HR of queried gene expression would be helpful in anticancer strategies. Two separate analyses were performed for the genes up-regulated in poor prognosis patients (HR > 1 by the Cox regression) and for those down-regulated in poor prognosis patients (HR < 1). From this analysis, we identified 12 DEGs whose expression levels are associated with significantly higher risk of tumor recurrence, and 4 genes have been reported to be related with survival or prognostic features. For instance, *MAP3K7* controls a variety of cell functions including transcription regulation and apoptosis through mediating the signaling transduction induced by TGFβ and bone morphogenetic protein (BMP) in a broad range of cancers [54–56].

KLHL21 interacts with Cullin3 and regulates mitosis in HeLa cells [57]. Unlike other family members, *KLHL21* regulates of the chromosomal passenger complex translocation at the onset of anaphase and is required for completion of cytokinesis [57]. It appears that *KLHL21* is the most promising gene among the 6 validated novel candidates. We identified for the first time that reduced expression of *KLHL21* is associated with decreased cell proliferation rate and invasion potential in HCC cells, although further research is required to fully illustrate the regulatory network and downstream targets of *KLHL21* in HCC development and progression.

Despite the significant body of literature describing predictive or prognostic mRNA profiles for cancer, only a small number are used in current oncology practice. Our study reveals novel biomarkers and molecular signatures related to HCC development and progression, making it possible to objectively evaluate the patient’s overall outcome and translate new molecular information into drug therapy.

## Conclusions

The significant outcomes from this study provide novel candidate genes for HCC on a genome-wide scale. Among them, *KLHL21* represents the most potential target for therapeutic intervention. Further prospective studies are warranted to seek inhibitors targeting *KLHL21* for the treatment of HCC.

## Additional files

Additional file 1: Table S1. The primers used in qRT-PCR analysis. (DOCX 12 kb)
Additional file 2: Figure S1. A flowchart for the datasets selection. + indicates more than. (TIF 914 kb)
Additional file 3: Table S2. The microarray gene expression datasets used in this study. (DOC 36 kb)
Additional file 4: Figure S2. Technical framework used in the meta-analysis. (TIF 3316 kb)
Additional file 5: Table S3. The dysregulated genes identified from this study. (XLSX 71 kb)
Additional file 6: Figure S3. Hierarchical clustering analysis of all dysregulated genes. (TIF 11970 kb)
Additional file 7: Figure S4. Directed acyclic graph of biological process of the dysregulated genes. The diagram represents the enriched GO sets containing at least 5 genes with a hypergeometric *p*-value less than 0.00001 (in red). (TIF 4339 kb)
Additional file 8: Figure S5. Directed acyclic graph of molecular function and cellular component of the dysregulated genes. The diagram represents the enriched GO sets containing at least 5 genes with a hypergeometric *p*-value less than 0.00001 (in red). (TIF 3401 kb)
Additional file 9: Figure S6. Forty genes were enriched in cell cycle pathway in KEGG analysis. (TIF 2764 kb)
Additional file 10: Table S4. The literature confirmation for the identified genes from our bioinformatics analysis. (DOCX 47 kb)

## Abbreviations

BP: Biological process
CC: Cellular component
DAVID: Database for Annotation, Visualization and Integrated Discover
DEGs: Different expression genes
FDR: False discovery rate
GO: Gene Oncology
HCC: Hepatocellular carcinoma
HR: Hazard ratio
IQR: Interquartile range
KEGG: Kyoto Encyclopedia of Genes and Genomes
MF: Molecular function
qRT-PCR: Quantitative RT-PCR
SAM: Significance analysis of microarray
WebGestalt: WEB-based GEne SeT AnaLysis Toolkit
