# Supplementary material for: KLHL21, a novel gene that contributes to the progression of hepatocellular carcinoma
Source: BMC Cancer. 2016 Oct 21;16:815. doi: 10.1186/s12885-016-2851-7 (PMC5073891; doi:10.1186/s12885-016-2851-7)
Supplement: Additional file 1: Table S1. — The primers used in qRT-PCR analysis. (DOCX 12 kb) [file 12885_2016_2851_MOESM1_ESM.docx]

| **Gene name** | **Primer sequences** |
| --- | --- |
| VPS45 | Forward: 5'-ggtttttgctgtgaagcagt-3' |
|  | Reverse: 5'-ctcagccacaacttcctgtt-3' |
| WIPI1 | Forward: 5'-gctgctgaggtggctgtc-3' |
|  | Reverse: 5'-ggacaagatgttgctggagt-3' |
| TTC1 | Forward: 5`-tgatcccaaaaatcagcatt-3` |
|  | Reverse: 5`-cattgatggccatttctttc-3` |
| GNB5 | Forward: 5`-agggaggttgccatctattc-3` |
|  | Reverse: 5`-acgcttagtgacctgtgagc-3` |
| IGBP1 | Forward: 5`-gttatcgcagctcgacttgt-3` |
|  | Reverse:  5`-cacagcagatttcattgcag-3` |
| KLHL2 | Forward: 5`-ctgtgtgtgcccaaggag-3` |
|  | Reverse:  5`-gtagcagtcgacagtgaccag-3` |
| KCNMA1 | Forward: 5`-ttctggctggaagtgaactc-3` |
|  | Reverse:  5`-cctgaattctccaccaaatg-3` |
| FCGRT | Forward: 5'-tgtcctggtattgggagaaa-3' |
|  | Reverse: 5'-tgcttgaggtcgaaattcat-3' |
| β-actin | Forward: 5'-cctcgcctttgccgatcc-3' |
|  | Reverse: 5'-ggatcttcatgaggtagtc-3' |

**Additional file 1: Table S1. The primers used in qRT-PCR analysis.**
