# Supplementary figures and images for: KLHL21, a novel gene that contributes to the progression of hepatocellular carcinoma
Source: BMC Cancer. 2016 Oct 21;16:815. doi: 10.1186/s12885-016-2851-7 (PMC5073891; doi:10.1186/s12885-016-2851-7)

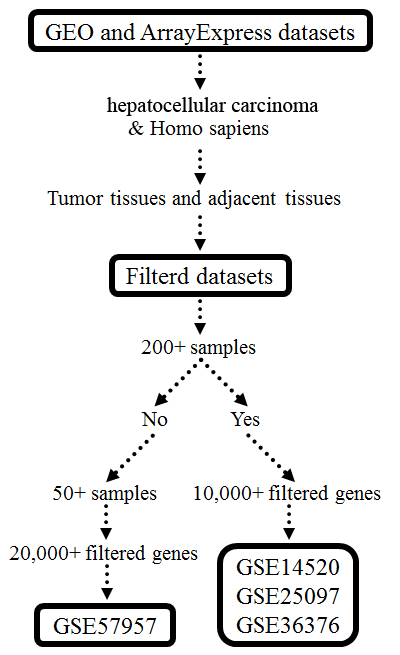

Supplement: Additional file 2: Figure S1. — A flowchart for the datasets selection. + indicates more than. (TIF 914 kb) [file 12885_2016_2851_MOESM2_ESM.tif]

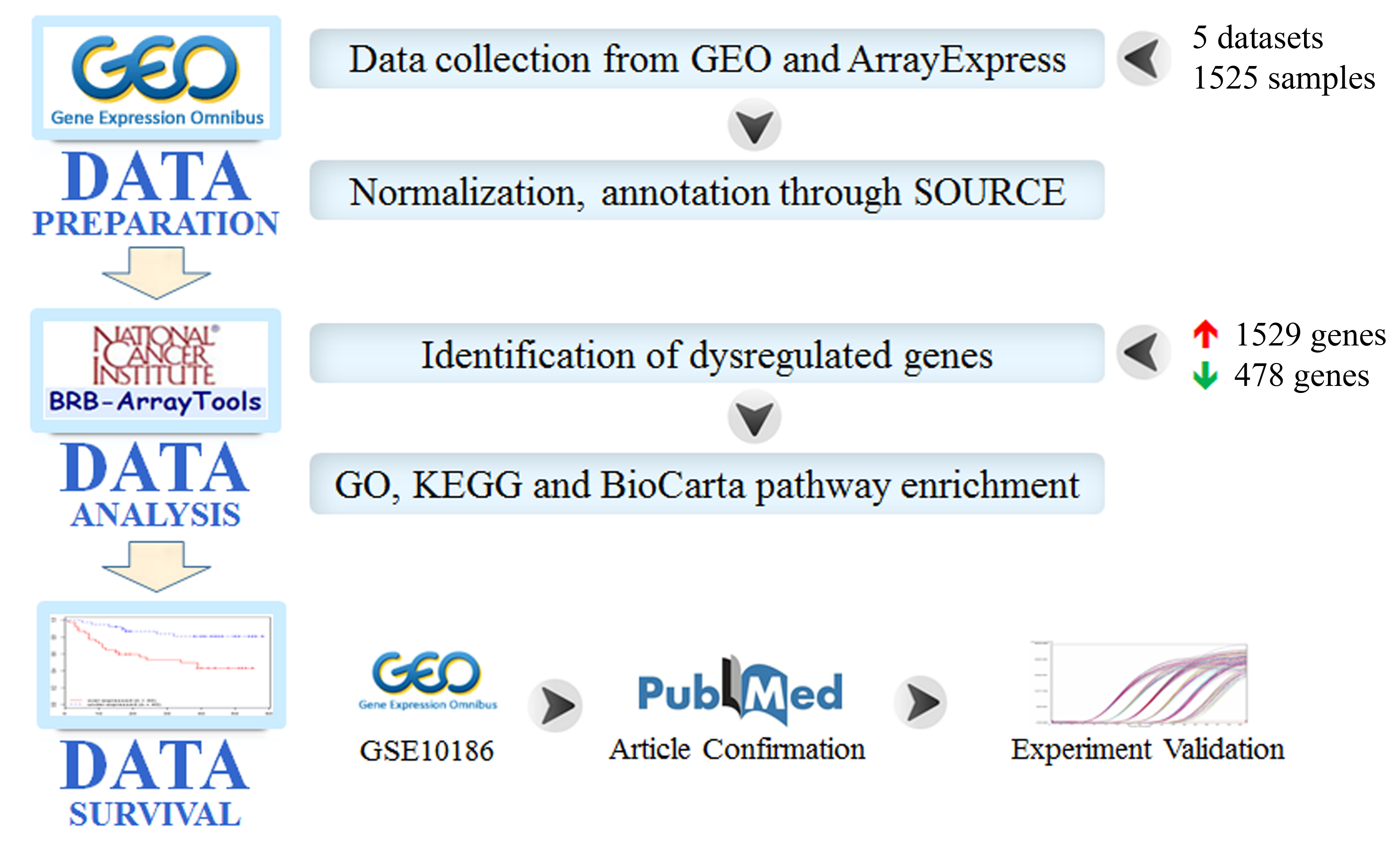

Supplement: Additional file 4: Figure S2. — Technical framework used in the meta-analysis. (TIF 3316 kb) [file 12885_2016_2851_MOESM4_ESM.tif]

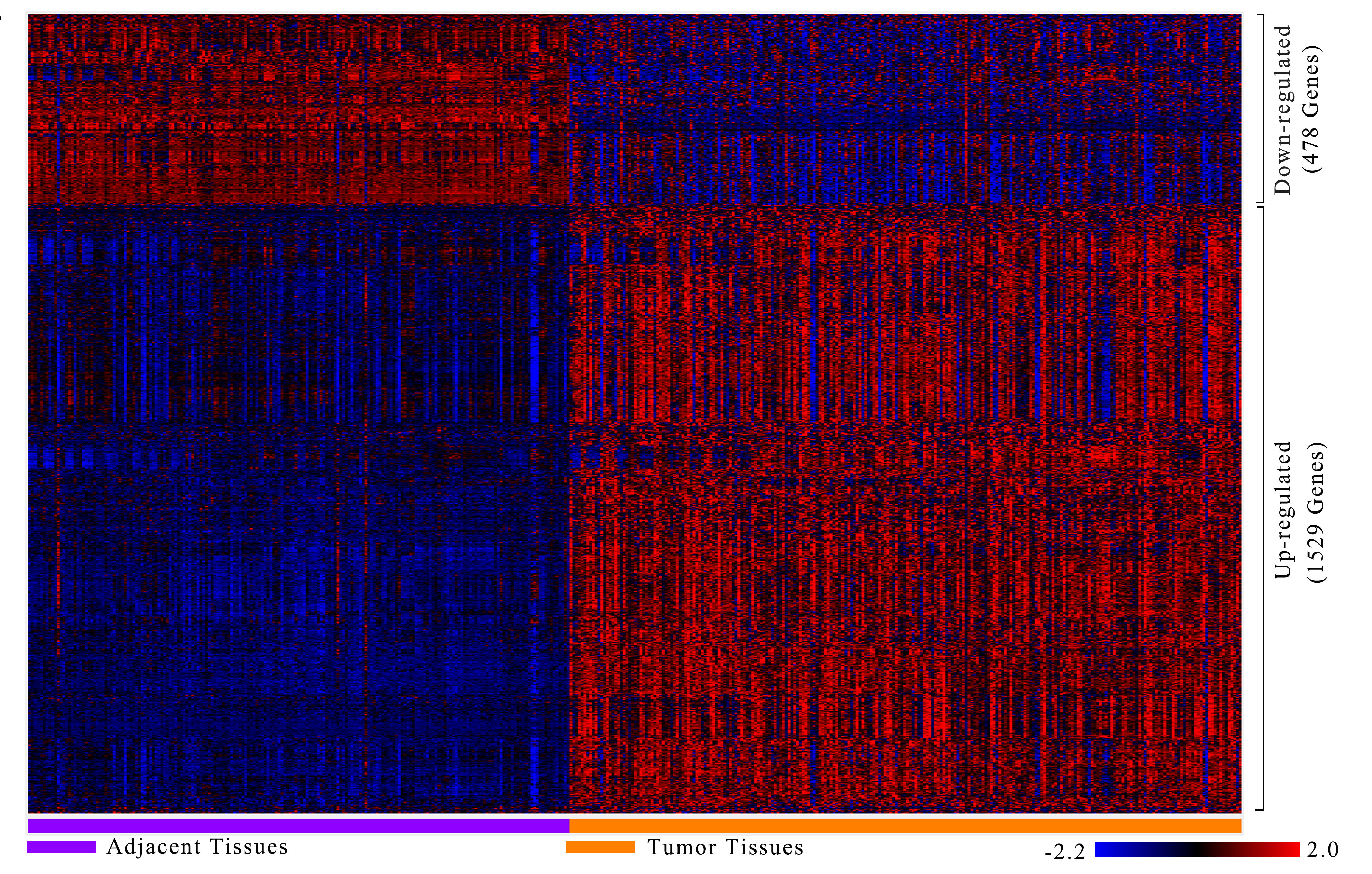

Supplement: Additional file 6: Figure S3. — Hierarchical clustering analysis of all dysregulated genes. (TIF 11970 kb) [file 12885_2016_2851_MOESM6_ESM.tif]

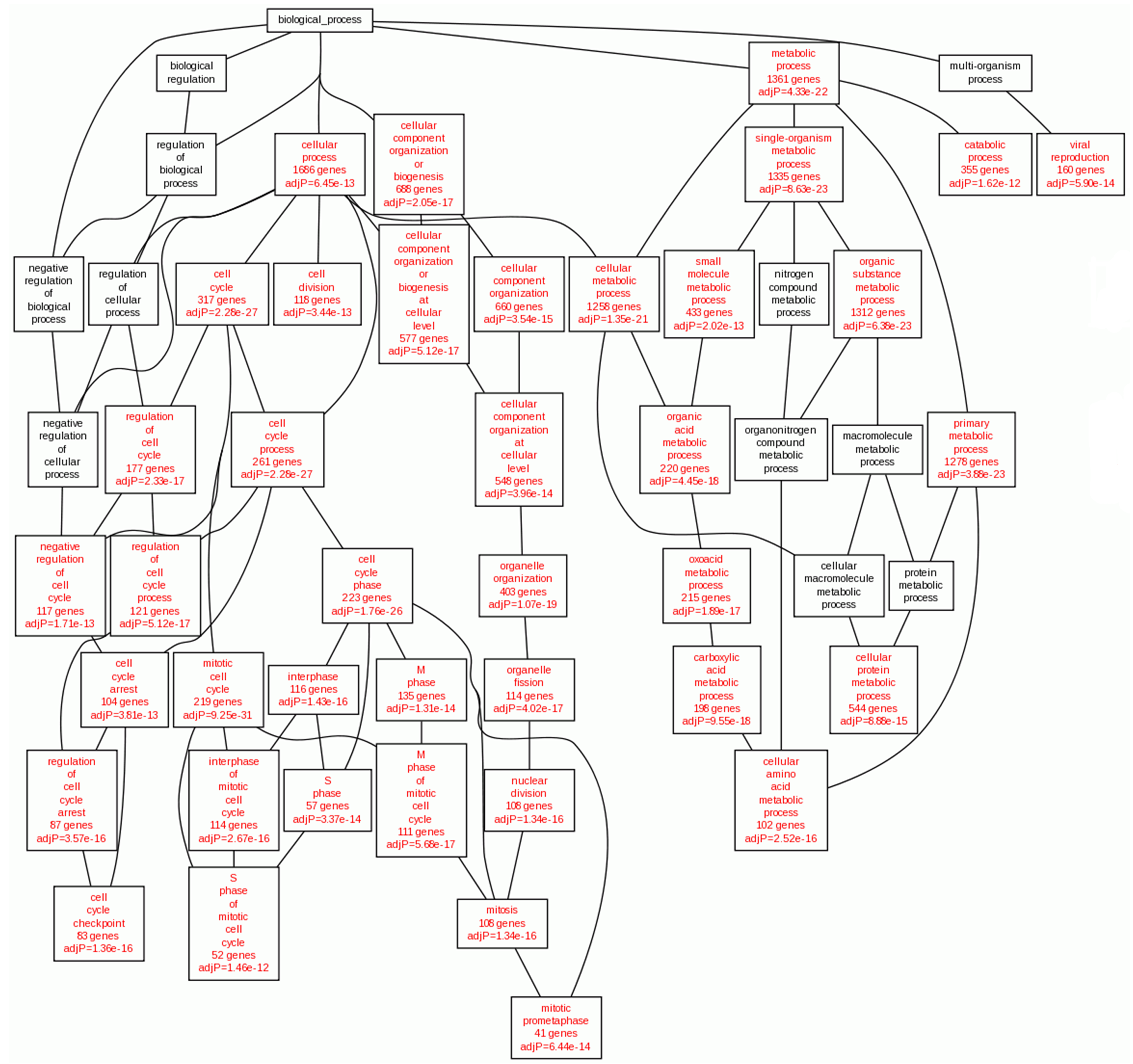

Supplement: Additional file 7: Figure S4. — Directed acyclic graph of biological process of the dysregulated genes. The diagram represents the enriched GO sets containing at least 5 genes with a hypergeometric p-value less than 0.00001 (in red). (TIF 4339 kb) [file 12885_2016_2851_MOESM7_ESM.tif]

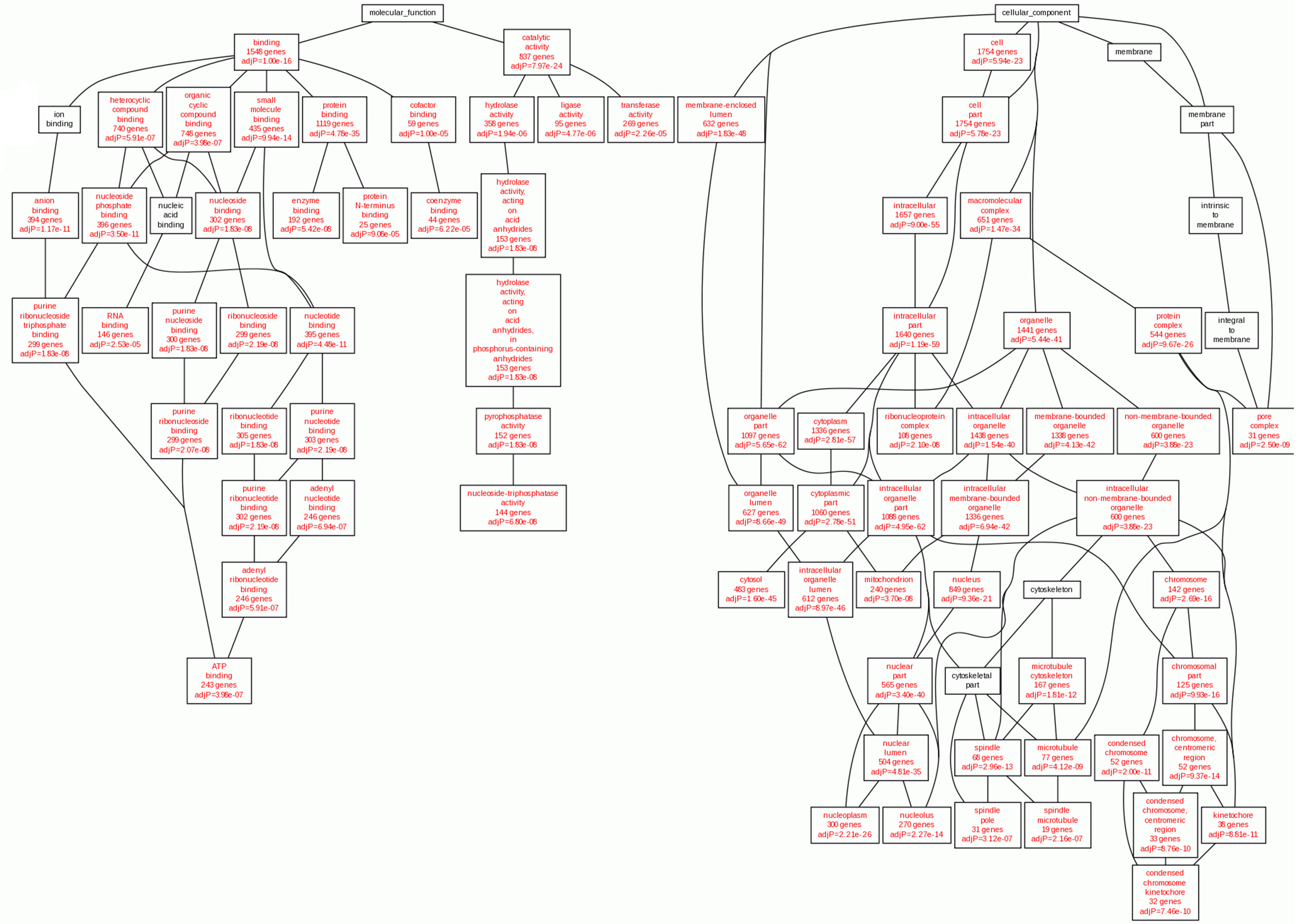

Supplement: Additional file 8: Figure S5. — Directed acyclic graph of molecular function and cellular component of the dysregulated genes. The diagram represents the enriched GO sets containing at least 5 genes with a hypergeometric p-value less than 0.00001 (in red). (TIF 3401 kb) [file 12885_2016_2851_MOESM8_ESM.tif]

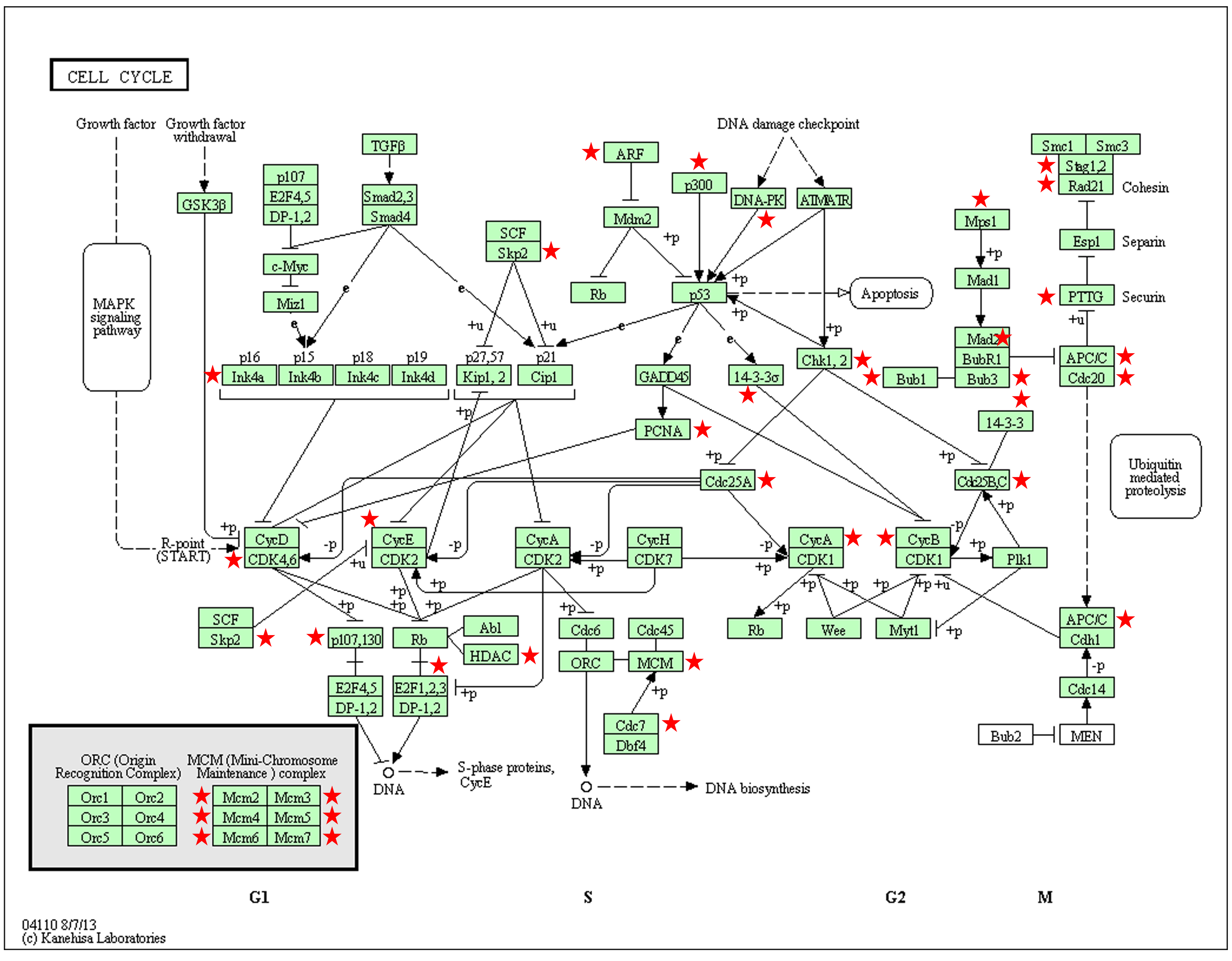

Supplement: Additional file 9: Figure S6. — Forty genes were enriched in cell cycle pathway in KEGG analysis. (TIF 2764 kb) [file 12885_2016_2851_MOESM9_ESM.tif]
