# Supplementary material for: KLHL21, a novel gene that contributes to the progression of hepatocellular carcinoma
Source: BMC Cancer. 2016 Oct 21;16:815. doi: 10.1186/s12885-016-2851-7 (PMC5073891; doi:10.1186/s12885-016-2851-7)
Supplement: Additional file 3: Table S2. — The microarray gene expression datasets used in this study. (DOC 36 kb) [file 12885_2016_2851_MOESM3_ESM.doc]

**Additional file 3: Table S2: The microarray gene expression datasets used in this**

**study.**

| **GEO ID** | **Platform** | **Number of**  **Filtered Genes** | **Samples**  **(Tumor : Adjacent)** |
| --- | --- | --- | --- |
| GSE14520^*^ [1, 2] | GPL3921  (Affymetrix HT Human Genome U133A Array) | 12,994 | 225 : 220 |
| GSE25097^*^ [3, 4] | GPL10687  (Rosetta/Merck Human RSTA Affymetrix 1.0 microarray, Custom CDF) | 20,442 | 268 : 243 |
| GSE36376^*^ [5] | GPL10558  (Illumina HumanHT-12 V4.0 expression beadchip) | 30,446 | 240 : 193 |
| GSE57957^*^ [6] | GPL10558  (Illumina HumanHT-12 V4.0 expression beadchip) | 31,413 | 37 : 37 |
| Total |  |  | 770 : 693 |
| GSE10186^#^ [7] | GPL5475  (Human 6k Transcriptionally Informative Gene Panel for DASL) | 5,729 | 62 : 0 |

* for meta-analysis; # for survival analysis

**References:**

1 Roessler S, Long EL, Budhu A, Chen Y, Zhao X, Ji J, Walker R, Jia HL, Ye QH, Qin LX, Tang ZY, He P, Hunter KW, Thorgeirsson SS, Meltzer PS, Wang XW: Integrative genomic identification of genes on 8p associated with hepatocellular carcinoma progression and patient survival. Gastroenterology 2012;142:957-966 e912.

2 Roessler S, Jia HL, Budhu A, Forgues M, Ye QH, Lee JS, Thorgeirsson SS, Sun Z, Tang ZY, Qin LX, Wang XW: A unique metastasis gene signature enables prediction of tumor relapse in early-stage hepatocellular carcinoma patients. Cancer research 2010;70:10202-10212.

3 Sung WK, Zheng H, Li S, Chen R, Liu X, Li Y, Lee NP, Lee WH, Ariyaratne PN, Tennakoon C, Mulawadi FH, Wong KF, Liu AM, Poon RT, Fan ST, Chan KL, Gong Z, Hu Y, Lin Z, Wang G, Zhang Q, Barber TD, Chou WC, Aggarwal A, Hao K, Zhou W, Zhang C, Hardwick J, Buser C, Xu J, Kan Z, Dai H, Mao M, Reinhard C, Wang J, Luk JM: Genome-wide survey of recurrent hbv integration in hepatocellular carcinoma. Nature genetics 2012;44:765-769.

4 Tung EK, Mak CK, Fatima S, Lo RC, Zhao H, Zhang C, Dai H, Poon RT, Yuen MF, Lai CL, Li JJ, Luk JM, Ng IO: Clinicopathological and prognostic significance of serum and tissue dickkopf-1 levels in human hepatocellular carcinoma. Liver international : official journal of the International Association for the Study of the Liver 2011;31:1494-1504.

5 Lim HY, Sohn I, Deng S, Lee J, Jung SH, Mao M, Xu J, Wang K, Shi S, Joh JW, Choi YL, Park CK: Prediction of disease-free survival in hepatocellular carcinoma by gene expression profiling. Annals of surgical oncology 2013;20:3747-3753.

6 Mah WC, Thurnherr T, Chow PK, Chung AY, Ooi LL, Toh HC, Teh BT, Saunthararajah Y, Lee CG: Methylation profiles reveal distinct subgroup of hepatocellular carcinoma patients with poor prognosis. PloS one 2014;9:e104158.

7 Hoshida Y, Nijman SM, Kobayashi M, Chan JA, Brunet JP, Chiang DY, Villanueva A, Newell P, Ikeda K, Hashimoto M, Watanabe G, Gabriel S, Friedman SL, Kumada H, Llovet JM, Golub TR: Integrative transcriptome analysis reveals common molecular subclasses of human hepatocellular carcinoma. Cancer research 2009;69:7385-7392.
