# Supplementary material for: KLHL21, a novel gene that contributes to the progression of hepatocellular carcinoma
Source: BMC Cancer. 2016 Oct 21;16:815. doi: 10.1186/s12885-016-2851-7 (PMC5073891; doi:10.1186/s12885-016-2851-7)
Supplement: Additional file 10: Table S4. — The literature confirmation for the identified genes from our bioinformatics analysis. (DOCX 47 kb) [file 12885_2016_2851_MOESM10_ESM.docx]

**Additional file 10: Table S4. The literature confirmation for the identified biomarkers from our bioinformatics analysis**

| **Gene**  **Symbol** | **Cancer Survival (PubMed Confirmation)** | | **Gene Function** |
| --- | --- | --- | --- |
|  | **Liver** | **Other Tissue Cancer** |  |
| HUWE1 |  | BTC [1] | A [2], I [3], P [3] |
| RBM3 |  | CRC [4-7], TC [8], GA [9], PC [10-12], BRC [13], ME [14, 15], OC [16], BTC[17] | A [18, 19], P [20] |
| MPV17 |  |  |  |
| NFE2L3 |  |  |  |
| PSMA4 |  | LC [21-23] | A [24], P [24] |
| ATP5G2 |  |  |  |
| GNB5 |  |  | I [25] |
| P2RX4 |  |  |  |
| TBCC |  | BTC [26] | P [26, 27] |
| WIPI1 |  |  | A [28, 29] |
| PSMD4 |  |  |  |
| GPAA1 |  |  | I [30], P [30, 31], T [30] |
| CKAP5 |  | LC [32], HNC [32] |  |
| CKAP4 | [33-35] |  | I [33-35], P [34, 36] |
| RHEB |  | CRC [37], etc | P [38, 39], A [37] |
| MYCN |  | NE [40, 41], etc |  |
| PSMD1 |  | HNC [42] |  |
| NOX4 |  | ME [43], PAC [44], LC [45], CRC [46] | A [43], I [47, 48], P [45, 48], T [49] |
| BID | [50] | CRC [51] | A [51-54] |
| FBL |  | BTC [55-57], LE [57, 58], PC [59] | P [59] |
| CCNF |  |  |  |
| SEMA4F |  |  | T [60], P [61] |
| KLHL21 |  |  |  |
| KIAA0196 |  |  | T [62] |
| COPS6 |  |  |  |
| ZKSCAN3 |  |  | I [63], T [64, 65] |
| RPS5 |  |  | T [66] |
| IGBP1 |  |  | A [67] |
| SNRPG |  |  |  |
| DBN1 |  | LC [68] | I [69] |
| MARS |  |  |  |
| ALDH18A1 |  |  | P [70] |
| NUP205 |  | LC [71] | P [71] |
| DDOST |  |  |  |
| MAP3K7 | [72] | PC [73-75], CRC [76, 77], PAC [78, 79], RC [80] | T [72], I [72] |
| ILF2 |  | LC [81], EC [82] | P [81] |
| CLN3 |  |  | P [83, 84], A [83, 84] |
| VPS45 |  |  |  |
| CCT4 |  |  | I [85, 86] |
| AGPS |  | BC [87, 88], ME [87, 88] | P [87-89], A [89, 90] |
| COPE |  |  |  |
| SSR2 |  |  | T [91], P [92] |
| RPL8 |  |  | P [93] |
| GPC3 | [94-98] | LC [99] | I [100, 101], P [100], T [101] |
| SCYL3 |  |  |  |
| CCL20 | [102-105] | LC [106, 107], CRC [108-110], PAC [111], GL [112] | I [102, 103, 113, 114], T [108, 115-118], P [102, 103] |
| GTF3C2 |  |  |  |
| SLC9A3R1 | [119] |  | A [120] |
| ZNF217 |  | GC [121], OC [122-124], BTC [125-127] | P [122], I [122, 128] |
| ATP6V1A |  |  |  |
| TTC1 |  |  |  |
| NCAPD2 |  |  | T [129] |
| NPC2 |  |  | A [130], P [130, 131], T [132] |
| SNAP23 |  |  | I [133] |
| BTN2A2 |  |  |  |
| APEX1 |  | LC [134, 135], GC [136, 137], CRC [138, 139], PC [140, 141], HNC [142], OS [143] | T [138], P [138], I [138] |
| SPTAN1 |  |  | T [144] |
| ACTR3 |  |  | I [145] |
| MYO5A |  | OSCC [146] |  |
| CBS | [147] | CRC [148, 149] | I [150-152], P [150-153], T [154] |
| CCT6B |  |  |  |
| IQGAP2 | [155, 156] | PAC [157] | T [158, 159] |
| GNAO1 |  | GL [160], GC [161] | P [161], A [161] |
| SDS |  |  |  |
| MPDZ |  |  |  |
| CTH |  | BRC [162] |  |
| KCNJ8 |  |  |  |
| SLC17A1 |  |  |  |
| KCNMA1 |  | PC [163] | P [164, 165], I [166] |
| PAPSS2 |  | PC [167] |  |
| UGP2 |  |  | A [168] |
| BCHE |  | RC [169], BRC [170], PC [171], HNC [172], OSCC [173] | P [174], T [175] |
| LIFR | [176] | ME [177], CRC [178] | I [176, 177, 179], P [180] |
| FCGRT |  |  |  |
| SLC27A2 |  |  | I [181] |
| TAT |  |  |  |
| CPS1 |  | CRC [182] | T [183] |
| BBOX1 |  |  |  |
| DCXR |  |  |  |

**Cancer Types:**

AS: Astrocytoma

BTC: Breast cancer

BRC: Bladder Cancer

CRC: Colorectal Cancer

HNC: Head and Neck Carcinoma

LC: Lung Cancer

ME: Melanoma

TC: Testicular Cancer

GA: Upper Gastrointestinal Adenocarcinoma

PC: Prostate cancer

NE: Neuroblastomas

PAC: Pancreatic Cancer

LA: Lung Adenocarcinoma

LE: Leukemia

EC: Esophageal Carcinoma

GL: Gliomas

GC: Gastric Cancer

OS: Osteosarcoma

OSCC: Oral Squamous Cell Carcinoma

ENC: Endometrial Cancer

PEC: Penile carcinoma

LT: lipomatous tumor

RC: Renal Carcinoma

CC: Cervical Cancer

**Prognostic Features:**

A: Apoptosis/Autophagy

I: Invasion/Migration/ Metastasis

D: Differentiation

P: Proliferation/Growth

T: Tumor progression

**References:**

1 Confalonieri S, Quarto M, Goisis G, Nuciforo P, Donzelli M, Jodice G, Pelosi G, Viale G, Pece S, Di Fiore PP: Alterations of ubiquitin ligases in human cancer and their association with the natural history of the tumor. Oncogene 2009;28:2959-2968.

2 Yi J, Lu G, Li L, Wang X, Cao L, Lin M, Zhang S, Shao G: DNA damage-induced activation of cul4b targets huwe1 for proteasomal degradation. Nucleic acids research 2015;43:4579-4590.

3 Vaughan L, Tan CT, Chapman A, Nonaka D, Mack NA, Smith D, Booton R, Hurlstone AF, Malliri A: Huwe1 ubiquitylates and degrades the rac activator tiam1 promoting cell-cell adhesion disassembly, migration, and invasion. Cell reports 2015;10:88-102.

4 Wang MJ, Ping J, Li Y, Adell G, Arbman G, Nodin B, Meng WJ, Zhang H, Yu YY, Wang C, Yang L, Zhou ZG, Sun XF: The prognostic factors and multiple biomarkers in young patients with colorectal cancer. Scientific reports 2015;5:10645.

5 Melling N, Simon R, Mirlacher M, Izbicki JR, Stahl P, Terracciano LM, Bokemeyer C, Sauter G, Marx AH: Loss of rna-binding motif protein 3 expression is associated with right-sided localization and poor prognosis in colorectal cancer. Histopathology 2015

6 Hjelm B, Brennan DJ, Zendehrokh N, Eberhard J, Nodin B, Gaber A, Ponten F, Johannesson H, Smaragdi K, Frantz C, Hober S, Johnson LB, Pahlman S, Jirstrom K, Uhlen M: High nuclear rbm3 expression is associated with an improved prognosis in colorectal cancer. Proteomics Clinical applications 2011;5:624-635.

7 Jonsson L, Gaber A, Ulmert D, Uhlen M, Bjartell A, Jirstrom K: High rbm3 expression in prostate cancer independently predicts a reduced risk of biochemical recurrence and disease progression. Diagnostic pathology 2011;6:91.

8 Olofsson SE, Nodin B, Gaber A, Eberhard J, Uhlen M, Jirstrom K, Jerkeman M: Low rbm3 protein expression correlates with clinical stage, prognostic classification and increased risk of treatment failure in testicular non-seminomatous germ cell cancer. PloS one 2015;10:e0121300.

9 Jonsson L, Hedner C, Gaber A, Korkocic D, Nodin B, Uhlen M, Eberhard J, Jirstrom K: High expression of rna-binding motif protein 3 in esophageal and gastric adenocarcinoma correlates with intestinal metaplasia-associated tumours and independently predicts a reduced risk of recurrence and death. Biomarker research 2014;2:11.

10 Grupp K, Wilking J, Prien K, Hube-Magg C, Sirma H, Simon R, Steurer S, Budaus L, Haese A, Izbicki J, Sauter G, Minner S, Schlomm T, Tsourlakis MC: High rna-binding motif protein 3 expression is an independent prognostic marker in operated prostate cancer and tightly linked to erg activation and pten deletions. European journal of cancer 2014;50:852-861.

11 Zeng Y, Wodzenski D, Gao D, Shiraishi T, Terada N, Li Y, Vander Griend DJ, Luo J, Kong C, Getzenberg RH, Kulkarni P: Stress-response protein rbm3 attenuates the stem-like properties of prostate cancer cells by interfering with cd44 variant splicing. Cancer research 2013;73:4123-4133.

12 Zeng Y, Kulkarni P, Inoue T, Getzenberg RH: Down-regulating cold shock protein genes impairs cancer cell survival and enhances chemosensitivity. Journal of cellular biochemistry 2009;107:179-188.

13 Boman K, Segersten U, Ahlgren G, Eberhard J, Uhlen M, Jirstrom K, Malmstrom PU: Decreased expression of rna-binding motif protein 3 correlates with tumour progression and poor prognosis in urothelial bladder cancer. BMC urology 2013;13:17.

14 Nodin B, Fridberg M, Jonsson L, Bergman J, Uhlen M, Jirstrom K: High mcm3 expression is an independent biomarker of poor prognosis and correlates with reduced rbm3 expression in a prospective cohort of malignant melanoma. Diagnostic pathology 2012;7:82.

15 Jonsson L, Bergman J, Nodin B, Manjer J, Ponten F, Uhlen M, Jirstrom K: Low rbm3 protein expression correlates with tumour progression and poor prognosis in malignant melanoma: An analysis of 215 cases from the malmo diet and cancer study. Journal of translational medicine 2011;9:114.

16 Ehlen A, Nodin B, Rexhepaj E, Brandstedt J, Uhlen M, Alvarado-Kristensson M, Ponten F, Brennan DJ, Jirstrom K: Rbm3-regulated genes promote DNA integrity and affect clinical outcome in epithelial ovarian cancer. Translational oncology 2011;4:212-221.

17 Jogi A, Brennan DJ, Ryden L, Magnusson K, Ferno M, Stal O, Borgquist S, Uhlen M, Landberg G, Pahlman S, Ponten F, Jirstrom K: Nuclear expression of the rna-binding protein rbm3 is associated with an improved clinical outcome in breast cancer. Modern pathology : an official journal of the United States and Canadian Academy of Pathology, Inc 2009;22:1564-1574.

18 Schneider J, Gomez-Esquer F, Diaz-Gil G, Torrejon R, Pollan M: Mrna expression of the putative antimetastatic gene brms1 and of apoptosis-related genes in breast cancer. Cancer genomics & proteomics 2011;8:195-197.

19 Martinez-Arribas F, Agudo D, Pollan M, Gomez-Esquer F, Diaz-Gil G, Lucas R, Schneider J: Positive correlation between the expression of x-chromosome rbm genes (rbmx, rbm3, rbm10) and the proapoptotic bax gene in human breast cancer. Journal of cellular biochemistry 2006;97:1275-1282.

20 Wellmann S, Truss M, Bruder E, Tornillo L, Zelmer A, Seeger K, Buhrer C: The rna-binding protein rbm3 is required for cell proliferation and protects against serum deprivation-induced cell death. Pediatric research 2010;67:35-41.

21 Wang T, Chen T, Thakur A, Liang Y, Gao L, Zhang S, Tian Y, Jin T, Liu JJ, Chen M: Association of psma4 polymorphisms with lung cancer susceptibility and response to cisplatin-based chemotherapy in a chinese han population. Clinical & translational oncology : official publication of the Federation of Spanish Oncology Societies and of the National Cancer Institute of Mexico 2015;17:564-569.

22 Hansen HM, Xiao Y, Rice T, Bracci PM, Wrensch MR, Sison JD, Chang JS, Smirnov IV, Patoka J, Seldin MF, Quesenberry CP, Kelsey KT, Wiencke JK: Fine mapping of chromosome 15q25.1 lung cancer susceptibility in african-americans. Human molecular genetics 2010;19:3652-3661.

23 Liu P, Vikis HG, Wang D, Lu Y, Wang Y, Schwartz AG, Pinney SM, Yang P, de Andrade M, Petersen GM, Wiest JS, Fain PR, Gazdar A, Gaba C, Rothschild H, Mandal D, Coons T, Lee J, Kupert E, Seminara D, Minna J, Bailey-Wilson JE, Wu X, Spitz MR, Eisen T, Houlston RS, Amos CI, Anderson MW, You M: Familial aggregation of common sequence variants on 15q24-25.1 in lung cancer. Journal of the National Cancer Institute 2008;100:1326-1330.

24 Liu Y, Liu P, Wen W, James MA, Wang Y, Bailey-Wilson JE, Amos CI, Pinney SM, Yang P, de Andrade M, Petersen GM, Wiest JS, Fain PR, Schwartz AG, Gazdar A, Gaba C, Rothschild H, Mandal D, Kupert E, Lee J, Seminara D, Minna J, Anderson MW, You M: Haplotype and cell proliferation analyses of candidate lung cancer susceptibility genes on chromosome 15q24-25.1. Cancer research 2009;69:7844-7850.

25 Fang LT, Lee S, Choi H, Kim HK, Jew G, Kang HC, Chen L, Jablons D, Kim IJ: Comprehensive genomic analyses of a metastatic colon cancer to the lung by whole exome sequencing and gene expression analysis. International journal of oncology 2014;44:211-221.

26 Hage-Sleiman R, Herveau S, Matera EL, Laurier JF, Dumontet C: Tubulin binding cofactor c (tbcc) suppresses tumor growth and enhances chemosensitivity in human breast cancer cells. BMC cancer 2010;10:135.

27 Hage-Sleiman R, Herveau S, Matera EL, Laurier JF, Dumontet C: Silencing of tubulin binding cofactor c modifies microtubule dynamics and cell cycle distribution and enhances sensitivity to gemcitabine in breast cancer cells. Molecular cancer therapeutics 2011;10:303-312.

28 Proikas-Cezanne T, Takacs Z, Donnes P, Kohlbacher O: Wipi proteins: Essential ptdins3p effectors at the nascent autophagosome. Journal of cell science 2015;128:207-217.

29 Tsuyuki S, Takabayashi M, Kawazu M, Kudo K, Watanabe A, Nagata Y, Kusama Y, Yoshida K: Detection of wipi1 mrna as an indicator of autophagosome formation. Autophagy 2014;10:497-513.

30 Wu G, Guo Z, Chatterjee A, Huang X, Rubin E, Wu F, Mambo E, Chang X, Osada M, Sook Kim M, Moon C, Califano JA, Ratovitski EA, Gollin SM, Sukumar S, Sidransky D, Trink B: Overexpression of glycosylphosphatidylinositol (gpi) transamidase subunits phosphatidylinositol glycan class t and/or gpi anchor attachment 1 induces tumorigenesis and contributes to invasion in human breast cancer. Cancer research 2006;66:9829-9836.

31 Ho JC, Cheung ST, Patil M, Chen X, Fan ST: Increased expression of glycosyl-phosphatidylinositol anchor attachment protein 1 (gpaa1) is associated with gene amplification in hepatocellular carcinoma. International journal of cancer Journal international du cancer 2006;119:1330-1337.

32 Martens-de Kemp SR, Nagel R, Stigter-van Walsum M, van der Meulen IH, van Beusechem VW, Braakhuis BJ, Brakenhoff RH: Functional genetic screens identify genes essential for tumor cell survival in head and neck and lung cancer. Clinical cancer research : an official journal of the American Association for Cancer Research 2013;19:1994-2003.

33 Li SX, Tang GS, Zhou DX, Pan YF, Tan YX, Zhang J, Zhang B, Ding ZW, Liu LJ, Jiang TY, Hu HP, Dong LW, Wang HY: Prognostic significance of cytoskeleton-associated membrane protein 4 and its palmitoyl acyltransferase dhhc2 in hepatocellular carcinoma. Cancer 2014;120:1520-1531.

34 Li SX, Liu LJ, Dong LW, Shi HG, Pan YF, Tan YX, Zhang J, Zhang B, Ding ZW, Jiang TY, Hu HP, Wang HY: Ckap4 inhibited growth and metastasis of hepatocellular carcinoma through regulating egfr signaling. Tumour biology : the journal of the International Society for Oncodevelopmental Biology and Medicine 2014;35:7999-8005.

35 Li MH, Dong LW, Li SX, Tang GS, Pan YF, Zhang J, Wang H, Zhou HB, Tan YX, Hu HP, Wang HY: Expression of cytoskeleton-associated protein 4 is related to lymphatic metastasis and indicates prognosis of intrahepatic cholangiocarcinoma patients after surgery resection. Cancer letters 2013;337:248-253.

36 Shahjee HM, Koch KR, Guo L, Zhang CO, Keay SK: Antiproliferative factor decreases akt phosphorylation and alters gene expression via ckap4 in t24 bladder carcinoma cells. Journal of experimental & clinical cancer research : CR 2010;29:160.

37 Campos T, Ziehe J, Palma M, Escobar D, Tapia JC, Pincheira R, Castro AF: Rheb promotes cancer cell survival through p27kip1-dependent activation of autophagy. Molecular carcinogenesis 2015

38 Fawal MA, Brandt M, Djouder N: Mcrs1 binds and couples rheb to amino acid-dependent mtorc1 activation. Developmental cell 2015;33:67-81.

39 Armijo ME, Campos T, Fuentes-Villalobos F, Palma ME, Pincheira R, Castro AF: Rheb signaling and tumorigenesis: Mtorc1 and new horizons. International journal of cancer Journal international du cancer 2015

40 Ramani P, Nash R, Sowa-Avugrah E, Rogers C: High levels of polo-like kinase 1 and phosphorylated translationally controlled tumor protein indicate poor prognosis in neuroblastomas. Journal of neuro-oncology 2015;125:103-111.

41 Beckers A, Van Peer G, Carter DR, Gartlgruber M, Herrmann C, Agarwal S, Helsmoortel HH, Althoff K, Molenaar JJ, Cheung BB, Schulte JH, Benoit Y, Shohet JM, Westermann F, Marshall GM, Vandesompele J, De Preter K, Speleman F: Mycn-driven regulatory mechanisms controlling lin28b in neuroblastoma. Cancer letters 2015;366:123-132.

42 Lagadec C, Vlashi E, Bhuta S, Lai C, Mischel P, Werner M, Henke M, Pajonk F: Tumor cells with low proteasome subunit expression predict overall survival in head and neck cancer patients. BMC cancer 2014;14:152.

43 Tanaka M, Miura Y, Numanami H, Karnan S, Ota A, Konishi H, Hosokawa Y, Hanyuda M: Inhibition of nadph oxidase 4 induces apoptosis in malignant mesothelioma: Role of reactive oxygen species. Oncology reports 2015;34:1726-1732.

44 Cheng G, Lanza-Jacoby S: Metformin decreases growth of pancreatic cancer cells by decreasing reactive oxygen species: Role of nox4. Biochemical and biophysical research communications 2015;465:41-46.

45 Li J, Lan T, Zhang C, Zeng C, Hou J, Yang Z, Zhang M, Liu J, Liu B: Reciprocal activation between il-6/stat3 and nox4/akt signalings promotes proliferation and survival of non-small cell lung cancer cells. Oncotarget 2015;6:1031-1048.

46 Bauer KM, Watts TN, Buechler S, Hummon AB: Proteomic and functional investigation of the colon cancer relapse-associated genes nox4 and itga3. Journal of proteome research 2014;13:4910-4918.

47 Kim HJ, Magesh V, Lee JJ, Kim S, Knaus UG, Lee KJ: Ubiquitin c-terminal hydrolase-l1 increases cancer cell invasion by modulating hydrogen peroxide generated via nadph oxidase 4. Oncotarget 2015;6:16287-16303.

48 Przybylska D, Mosieniak G: [the role of nadph oxidase nox4 in regulation of proliferation, senescence and differentiation of the cells]. Postepy biochemii 2014;60:69-76.

49 Liu ZM, Tseng HY, Tsai HW, Su FC, Huang HS: Transforming growth factor beta-interacting factor-induced malignant progression of hepatocellular carcinoma cells depends on superoxide production from nox4. Free radical biology & medicine 2015;84:54-64.

50 Orlik J, Schungel S, Buitrago-Molina LE, Marhenke S, Geffers R, Endig J, Lobschat K, Rossler S, Goeppert B, Manns MP, Gross A, Vogel A: The bh3-only protein bid impairs the p38-mediated stress response and promotes hepatocarcinogenesis during chronic liver injury in mice. Hepatology 2015;62:816-828.

51 Leibowitz B, Qiu W, Buchanan ME, Zou F, Vernon P, Moyer MP, Yin XM, Schoen RE, Yu J, Zhang L: Bid mediates selective killing of apc-deficient cells in intestinal tumor suppression by nonsteroidal antiinflammatory drugs. Proceedings of the National Academy of Sciences of the United States of America 2014;111:16520-16525.

52 Orzechowska EJ, Girstun A, Staron K, Trzcinska-Danielewicz J: Synergy of bid with doxorubicin in the killing of cancer cells. Oncology reports 2015;33:2143-2150.

53 Prakasam A, Ghose S, Oleinik NV, Bethard JR, Peterson YK, Krupenko NI, Krupenko SA: Jnk1/2 regulate bid by direct phosphorylation at thr59 in response to aldh1l1. Cell Death Dis 2014;5:e1358.

54 Barathan M, Mariappan V, Shankar EM, Abdullah BJ, Goh KL, Vadivelu J: Hypericin-photodynamic therapy leads to interleukin-6 secretion by hepg2 cells and their apoptosis via recruitment of bh3 interacting-domain death agonist and caspases. Cell Death Dis 2013;4:e697.

55 Marcel V, Ghayad SE, Belin S, Therizols G, Morel AP, Solano-Gonzalez E, Vendrell JA, Hacot S, Mertani HC, Albaret MA, Bourdon JC, Jordan L, Thompson A, Tafer Y, Cong R, Bouvet P, Saurin JC, Catez F, Prats AC, Puisieux A, Diaz JJ: P53 acts as a safeguard of translational control by regulating fibrillarin and rrna methylation in cancer. Cancer Cell 2013;24:318-330.

56 Su H, Xu T, Ganapathy S, Shadfan M, Long M, Huang TH, Thompson I, Yuan ZM: Elevated snorna biogenesis is essential in breast cancer. Oncogene 2014;33:1348-1358.

57 Cao Y, Zhao Y, Yu Y, Wang Y, Zhang M, Zhang W, Wang J: Granulocyte-macrophage colony-stimulating factor induces the differentiation of murine erythroleukaemia cells into dendritic cells. Immunology 1998;95:141-147.

58 He A, Zhang W, Xu K, Wang J, Yang Y, Chao X: Anti-tumor immune responses in immune-reconstituted mice injected with a tumor vaccine. Med Oncol 2012;29:2261-2269.

59 Koh CM, Gurel B, Sutcliffe S, Aryee MJ, Schultz D, Iwata T, Uemura M, Zeller KI, Anele U, Zheng Q, Hicks JL, Nelson WG, Dang CV, Yegnasubramanian S, De Marzo AM: Alterations in nucleolar structure and gene expression programs in prostatic neoplasia are driven by the myc oncogene. Am J Pathol 2011;178:1824-1834.

60 Gabrovska PN, Smith RA, Tiang T, Weinstein SR, Haupt LM, Griffiths LR: Semaphorin-plexin signalling genes associated with human breast tumourigenesis. Gene 2011;489:63-69.

61 Parrinello S, Noon LA, Harrisingh MC, Wingfield Digby P, Rosenberg LH, Cremona CA, Echave P, Flanagan AM, Parada LF, Lloyd AC: Nf1 loss disrupts schwann cell-axonal interactions: A novel role for semaphorin 4f. Genes Dev 2008;22:3335-3348.

62 van Duin M, van Marion R, Vissers K, Watson JE, van Weerden WM, Schroder FH, Hop WC, van der Kwast TH, Collins C, van Dekken H: High-resolution array comparative genomic hybridization of chromosome arm 8q: Evaluation of genetic progression markers for prostate cancer. Genes, chromosomes & cancer 2005;44:438-449.

63 Zhang X, Jing Y, Qin Y, Hunsucker S, Meng H, Sui J, Jiang Y, Gao L, An G, Yang N, Orlowski RZ, Yang L: The zinc finger transcription factor zkscan3 promotes prostate cancer cell migration. The international journal of biochemistry & cell biology 2012;44:1166-1173.

64 Yang L, Zhang L, Wu Q, Boyd DD: Unbiased screening for transcriptional targets of zkscan3 identifies integrin beta 4 and vascular endothelial growth factor as downstream targets. J Biol Chem 2008;283:35295-35304.

65 Yang L, Hamilton SR, Sood A, Kuwai T, Ellis L, Sanguino A, Lopez-Berestein G, Boyd DD: The previously undescribed zkscan3 (znf306) is a novel "driver" of colorectal cancer progression. Cancer research 2008;68:4321-4330.

66 Bandres E, Malumbres R, Cubedo E, Honorato B, Zarate R, Labarga A, Gabisu U, Sola JJ, Garcia-Foncillas J: A gene signature of 8 genes could identify the risk of recurrence and progression in dukes' b colon cancer patients. Oncology reports 2007;17:1089-1094.

67 Li D, Sakashita S, Morishita Y, Kano J, Shiba A, Sato T, Noguchi M: Binding of lactoferrin to igbp1 triggers apoptosis in a lung adenocarcinoma cell line. Anticancer Res 2011;31:529-534.

68 Mitra R, Lee J, Jo J, Milani M, McClintick JN, Edenberg HJ, Kesler KA, Rieger KM, Badve S, Cummings OW, Mohiuddin A, Thomas DG, Luo X, Juliar BE, Li L, Mesaros C, Blair IA, Srirangam A, Kratzke RA, McDonald CJ, Kim J, Potter DA: Prediction of postoperative recurrence-free survival in non-small cell lung cancer by using an internationally validated gene expression model. Clinical cancer research : an official journal of the American Association for Cancer Research 2011;17:2934-2946.

69 Lin Q, Tan HT, Lim TK, Khoo A, Lim KH, Chung MC: Itraq analysis of colorectal cancer cell lines suggests drebrin (dbn1) is overexpressed during liver metastasis. Proteomics 2014;14:1434-1443.

70 Kardos GR, Wastyk HC, Robertson GP: Disruption of proline synthesis in melanoma inhibits protein production mediated by the gcn2 pathway. Molecular cancer research : MCR 2015;13:1408-1420.

71 Fujitomo T, Daigo Y, Matsuda K, Ueda K, Nakamura Y: Critical function for nuclear envelope protein tmem209 in human pulmonary carcinogenesis. Cancer research 2012;72:4110-4118.

72 Roh YS, Song J, Seki E: Tak1 regulates hepatic cell survival and carcinogenesis. J Gastroenterol 2014;49:185-194.

73 Rodrigues LU, Rider L, Nieto C, Romero L, Karimpour-Fard A, Loda M, Lucia MS, Wu M, Shi L, Cimic A, Sirintrapun SJ, Nolley R, Pac C, Chen H, Peehl DM, Xu J, Liu W, Costello JC, Cramer SD: Coordinate loss of map3k7 and chd1 promotes aggressive prostate cancer. Cancer research 2015;75:1021-1034.

74 Wu M, Shi L, Cimic A, Romero L, Sui G, Lees CJ, Cline JM, Seals DF, Sirintrapun JS, McCoy TP, Liu W, Kim JW, Hawkins GA, Peehl DM, Xu J, Cramer SD: Suppression of tak1 promotes prostate tumorigenesis. Cancer research 2012;72:2833-2843.

75 Liu W, Chang BL, Cramer S, Koty PP, Li T, Sun J, Turner AR, Von Kap-Herr C, Bobby P, Rao J, Zheng SL, Isaacs WB, Xu J: Deletion of a small consensus region at 6q15, including the map3k7 gene, is significantly associated with high-grade prostate cancers. Clinical cancer research : an official journal of the American Association for Cancer Research 2007;13:5028-5033.

76 Singh A, Sweeney MF, Yu M, Burger A, Greninger P, Benes C, Haber DA, Settleman J: Tak1 inhibition promotes apoptosis in kras-dependent colon cancers. Cell 2012;148:639-650.

77 Slattery ML, Lundgreen A, Bondurant KL, Wolff RK: Tumor necrosis factor-related genes and colon and rectal cancer. International journal of molecular epidemiology and genetics 2011;2:328-338.

78 Giroux V, Dagorn JC, Iovanna JL: A review of kinases implicated in pancreatic cancer. Pancreatology 2009;9:738-754.

79 Giroux V, Iovanna JL, Garcia S, Dagorn JC: Combined inhibition of pak7, map3k7 and ck2alpha kinases inhibits the growth of miapaca2 pancreatic cancer cell xenografts. Cancer gene therapy 2009;16:731-740.

80 Wei C, Lai YQ, Li XX, Ye JX: Tgf-beta-activated kinase-1: A potential prognostic marker for clear cell renal cell carcinoma. Asian Pacific journal of cancer prevention : APJCP 2013;14:315-320.

81 Ni T, Mao G, Xue Q, Liu Y, Chen B, Cui X, Lv L, Jia L, Wang Y, Ji L: Upregulated expression of ilf2 in non-small cell lung cancer is associated with tumor cell proliferation and poor prognosis. J Mol Histol 2015;46:325-335.

82 Ni S, Zhu J, Zhang J, Zhang S, Li M, Ni R, Liu J, Qiu H, Chen W, Wang H, Guo W: Expression and clinical role of nf45 as a novel cell cycle protein in esophageal squamous cell carcinoma (escc). Tumour biology : the journal of the International Society for Oncodevelopmental Biology and Medicine 2015;36:747-756.

83 Mao D, Che J, Han S, Zhao H, Zhu Y, Zhu H: Rnai-mediated knockdown of the cln3 gene inhibits proliferation and promotes apoptosis in drug-resistant ovarian cancer cells. Molecular medicine reports 2015;12:6635-6641.

84 Zhu X, Huang Z, Chen Y, Zhou J, Hu S, Zhi Q, Song S, Wang Y, Wan D, Gu W, Zhou H, Zhang B, Cao W, He S: Effect of cln3 silencing by rna interference on the proliferation and apoptosis of human colorectal cancer cells. Biomed Pharmacother 2014;68:253-258.

85 Wang X, Li M, Wang Z, Han S, Tang X, Ge Y, Zhou L, Zhou C, Yuan Q, Yang M: Silencing of long noncoding rna malat1 by mir-101 and mir-217 inhibits proliferation, migration, and invasion of esophageal squamous cell carcinoma cells. J Biol Chem 2015;290:3925-3935.

86 Tano K, Mizuno R, Okada T, Rakwal R, Shibato J, Masuo Y, Ijiri K, Akimitsu N: Malat-1 enhances cell motility of lung adenocarcinoma cells by influencing the expression of motility-related genes. Febs Lett 2010;584:4575-4580.

87 Benjamin DI, Cozzo A, Ji X, Roberts LS, Louie SM, Mulvihill MM, Luo K, Nomura DK: Ether lipid generating enzyme agps alters the balance of structural and signaling lipids to fuel cancer pathogenicity. Proceedings of the National Academy of Sciences of the United States of America 2013;110:14912-14917.

88 Piano V, Benjamin DI, Valente S, Nenci S, Marrocco B, Mai A, Aliverti A, Nomura DK, Mattevi A: Discovery of inhibitors for the ether lipid-generating enzyme agps as anti-cancer agents. ACS chemical biology 2015;10:2589-2597.

89 Zhu Y, Liu XJ, Yang P, Zhao M, Lv LX, Zhang GD, Wang Q, Zhang L: Alkylglyceronephosphate synthase (agps) alters lipid signaling pathways and supports chemotherapy resistance of glioma and hepatic carcinoma cell lines. Asian Pacific journal of cancer prevention : APJCP 2014;15:3219-3226.

90 Ishaq M, Kumar S, Varinli H, Han ZJ, Rider AE, Evans MD, Murphy AB, Ostrikov K: Atmospheric gas plasma-induced ros production activates tnf-ask1 pathway for the induction of melanoma cancer cell apoptosis. Molecular biology of the cell 2014;25:1523-1531.

91 Chano T, Mori K, Scotlandi K, Benini S, Lapucci C, Manara MC, Serra M, Picci P, Okabe H, Baldini N: Differentially expressed genes in multidrug resistant variants of u-2 os human osteosarcoma cells. Oncology reports 2004;11:1257-1263.

92 Fisher WE, Wu Y, Amaya F, Berger DH: Somatostatin receptor subtype 2 gene therapy inhibits pancreatic cancer in vitro. J Surg Res 2002;105:58-64.

93 Swoboda RK, Somasundaram R, Caputo L, Ochoa EM, Gimotty PA, Marincola FM, Van Belle P, Barth S, Elder D, Guerry D, Czerniecki B, Schuchter L, Vonderheide RH, Herlyn D: Shared mhc class ii-dependent melanoma ribosomal protein l8 identified by phage display. Cancer research 2007;67:3555-3559.

94 Wu Y, Liu H, Weng H, Zhang X, Li P, Fan CL, Li B, Dong PL, Li L, Dooley S, Ding HG: Glypican-3 promotes epithelial-mesenchymal transition of hepatocellular carcinoma cells through erk signaling pathway. International journal of oncology 2015;46:1275-1285.

95 Qi XH, Wu D, Cui HX, Ma N, Su J, Wang YT, Jiang YH: Silencing of the glypican-3 gene affects the biological behavior of human hepatocellular carcinoma cells. Molecular medicine reports 2014;10:3177-3184.

96 Xiao WK, Qi CY, Chen D, Li SQ, Fu SJ, Peng BG, Liang LJ: Prognostic significance of glypican-3 in hepatocellular carcinoma: A meta-analysis. BMC cancer 2014;14:104.

97 Feng M, Ho M: Glypican-3 antibodies: A new therapeutic target for liver cancer. Febs Lett 2014;588:377-382.

98 Fu SJ, Qi CY, Xiao WK, Li SQ, Peng BG, Liang LJ: Glypican-3 is a potential prognostic biomarker for hepatocellular carcinoma after curative resection. Surgery 2013;154:536-544.

99 Yu X, Li Y, Chen SW, Shi Y, Xu F: Differential expression of glypican-3 (gpc3) in lung squamous cell carcinoma and lung adenocarcinoma and its clinical significance. Genetics and molecular research : GMR 2015;14:10185-10192.

100 Gao W, Kim H, Ho M: Human monoclonal antibody targeting the heparan sulfate chains of glypican-3 inhibits hgf-mediated migration and motility of hepatocellular carcinoma cells. PloS one 2015;10:e0137664.

101 Liu Y, Zheng D, Liu M, Bai J, Zhou X, Gong B, Lu J, Zhang Y, Huang H, Luo W, Huang G: Downregulation of glypican-3 expression increases migration, invasion, and tumorigenicity of human ovarian cancer cells. Tumour biology : the journal of the International Society for Oncodevelopmental Biology and Medicine 2015;36:7997-8006.

102 Hou KZ, Fu ZQ, Gong H: Chemokine ligand 20 enhances progression of hepatocellular carcinoma via epithelial-mesenchymal transition. World J Gastroenterol 2015;21:475-483.

103 Du D, Liu Y, Qian H, Zhang B, Tang X, Zhang T, Liu W: The effects of the ccr6/ccl20 biological axis on the invasion and metastasis of hepatocellular carcinoma. Int J Mol Sci 2014;15:6441-6452.

104 Ding X, Wang K, Wang H, Zhang G, Liu Y, Yang Q, Chen W, Hu S: High expression of ccl20 is associated with poor prognosis in patients with hepatocellular carcinoma after curative resection. J Gastrointest Surg 2012;16:828-836.

105 Chen KJ, Lin SZ, Zhou L, Xie HY, Zhou WH, Taki-Eldin A, Zheng SS: Selective recruitment of regulatory t cell through ccr6-ccl20 in hepatocellular carcinoma fosters tumor progression and predicts poor prognosis. PloS one 2011;6:e24671.

106 Wang GZ, Cheng X, Li XC, Liu YQ, Wang XQ, Shi X, Wang ZY, Guo YQ, Wen ZS, Huang YC, Zhou GB: Tobacco smoke induces production of chemokine ccl20 to promote lung cancer. Cancer letters 2015;363:60-70.

107 Yang G, Li H, Yao Y, Xu F, Bao Z, Zhou J: Treg/th17 imbalance in malignant pleural effusion partially predicts poor prognosis. Oncology reports 2015;33:478-484.

108 Cheng XS, Li YF, Tan J, Sun B, Xiao YC, Fang XB, Zhang XF, Li Q, Dong JH, Li M, Qian HH, Yin ZF, Yang ZB: Ccl20 and cxcl8 synergize to promote progression and poor survival outcome in patients with colorectal cancer by collaborative induction of the epithelial-mesenchymal transition. Cancer letters 2014;348:77-87.

109 Frick VO, Rubie C, Kolsch K, Wagner M, Ghadjar P, Graeber S, Glanemann M: Ccr6/ccl20 chemokine expression profile in distinct colorectal malignancies. Scandinavian journal of immunology 2013;78:298-305.

110 Ghadjar P, Rubie C, Aebersold DM, Keilholz U: The chemokine ccl20 and its receptor ccr6 in human malignancy with focus on colorectal cancer. International journal of cancer Journal international du cancer 2009;125:741-745.

111 Klemm C, Dommisch H, Goke F, Kreppel M, Jepsen S, Rolf F, Dommisch K, Perner S, Standop J: Expression profiles for 14-3-3 zeta and ccl20 in pancreatic cancer and chronic pancreatitis. Pathology, research and practice 2014;210:335-341.

112 Wang L, Qin H, Li L, Zhang Y, Tu Y, Feng F, Ji P, Zhang J, Li G, Zhao Z, Gao G: Overexpression of ccl20 and its receptor ccr6 predicts poor clinical prognosis in human gliomas. Med Oncol 2012;29:3491-3497.

113 Chen CH, Chuang HC, Lin YT, Fang FM, Huang CC, Chen CM, Lu H, Chien CY: Circulating cd105 shows significant impact in patients of oral cancer and promotes malignancy of cancer cells via ccl20. Tumour biology : the journal of the International Society for Oncodevelopmental Biology and Medicine 2015

114 Zeng W, Chang H, Ma M, Li Y: Ccl20/ccr6 promotes the invasion and migration of thyroid cancer cells via nf-kappa b signaling-induced mmp-3 production. Exp Mol Pathol 2014;97:184-190.

115 Zhang J, Zhu D, Lv Q, Yi Y, Li F, Zhang W: The key role of astrocyte elevated gene-1 in ccr6-induced emt in cervical cancer. Tumour biology : the journal of the International Society for Oncodevelopmental Biology and Medicine 2015;36:9763-9767.

116 Han G, Wu D, Yang Y, Li Z, Zhang J, Li C: Crkl meditates ccl20/ccr6-induced emt in gastric cancer. Cytokine 2015;76:163-169.

117 Chin CC, Chen CN, Kuo HC, Shi CS, Hsieh MC, Kuo YH, Tung SY, Lee KF, Huang WS: Interleukin-17 induces cc chemokine receptor 6 expression and cell migration in colorectal cancer cells. Journal of cellular physiology 2015;230:1430-1437.

118 Sligh J, Janda J, Jandova J: Mutations in balb mitochondrial DNA induce ccl20 up-regulation promoting tumorigenic phenotypes. Mutation research 2014;769:49-58.

119 Zhang J, Wen B, Cong W, Chen L, Jiang J, Pan W, He J, Zhu Z: [association of chromosome 17q copy number variation with overall survival of patients with hepatocellular carcinoma and screening of potential target genes]. Zhonghua yi xue yi chuan xue za zhi = Zhonghua yixue yichuanxue zazhi = Chinese journal of medical genetics 2015;32:615-619.

120 Liu H, Ma Y, He HW, Wang JP, Jiang JD, Shao RG: Slc9a3r1 stimulates autophagy via becn1 stabilization in breast cancer cells. Autophagy 2015:0.

121 Shida A, Fujioka S, Kurihara H, Ishibashi Y, Mitsumori N, Omura N, Yanaga K: Prognostic significance of znf217 expression in gastric carcinoma. Anticancer Res 2014;34:4813-4817.

122 Li J, Song LL, Qiu YW, Yin AL, Zhong M: Znf217 is associated with poor prognosis and enhances proliferation and metastasis in ovarian cancer. International journal of clinical and experimental pathology 2014;7:3038-3047.

123 Li J, Song LL, Zhong M: [znf217 expression correlates with the biological behavior of human ovarian cancer cells]. Zhonghua zhong liu za zhi [Chinese journal of oncology] 2013;35:170-174.

124 Sun G, Zhou J, Yin A, Ding Y, Zhong M: Silencing of znf217 gene influences the biological behavior of a human ovarian cancer cell line. International journal of oncology 2008;32:1065-1071.

125 Littlepage LE, Adler AS, Kouros-Mehr H, Huang G, Chou J, Krig SR, Griffith OL, Korkola JE, Qu K, Lawson DA, Xue Q, Sternlicht MD, Dijkgraaf GJ, Yaswen P, Rugo HS, Sweeney CA, Collins CC, Gray JW, Chang HY, Werb Z: The transcription factor znf217 is a prognostic biomarker and therapeutic target during breast cancer progression. Cancer discovery 2012;2:638-651.

126 Vendrell JA, Thollet A, Nguyen NT, Ghayad SE, Vinot S, Bieche I, Grisard E, Josserand V, Coll JL, Roux P, Corbo L, Treilleux I, Rimokh R, Cohen PA: Znf217 is a marker of poor prognosis in breast cancer that drives epithelial-mesenchymal transition and invasion. Cancer research 2012;72:3593-3606.

127 Krig SR, Miller JK, Frietze S, Beckett LA, Neve RM, Farnham PJ, Yaswen PI, Sweeney CA: Znf217, a candidate breast cancer oncogene amplified at 20q13, regulates expression of the erbb3 receptor tyrosine kinase in breast cancer cells. Oncogene 2010;29:5500-5510.

128 Bai WD, Ye XM, Zhang MY, Zhu HY, Xi WJ, Huang X, Zhao J, Gu B, Zheng GX, Yang AG, Jia LT: Mir-200c suppresses tgf-beta signaling and counteracts trastuzumab resistance and metastasis by targeting znf217 and zeb1 in breast cancer. International journal of cancer Journal international du cancer 2014;135:1356-1368.

129 Emmanuel C, Gava N, Kennedy C, Balleine RL, Sharma R, Wain G, Brand A, Hogg R, Etemadmoghadam D, George J, Australian Ovarian Cancer Study G, Birrer MJ, Clarke CL, Chenevix-Trench G, Bowtell DD, Harnett PR, deFazio A: Comparison of expression profiles in ovarian epithelium in vivo and ovarian cancer identifies novel candidate genes involved in disease pathogenesis. PloS one 2011;6:e17617.

130 Adachi T, Matsumoto Y, Inagaki Y, Sekimizu K: Niemann-pick disease type c2 protein induces autophagy and inhibits growth in fm3a breast cancer cells. Drug discoveries & therapeutics 2015;9:282-288.

131 Liao YJ, Fang CC, Yen CH, Hsu SM, Wang CK, Huang SF, Liang YC, Lin YY, Chu YT, Arthur Chen YM: Niemann-pick type c2 protein regulates liver cancer progression via modulating erk1/2 pathway: Clinicopathological correlations and therapeutical implications. International journal of cancer Journal international du cancer 2015;137:1341-1351.

132 Liao YJ, Lin MW, Yen CH, Lin YT, Wang CK, Huang SF, Chen KH, Yang CP, Chen TL, Hou MF, Arthur Chen YM: Characterization of niemann-pick type c2 protein expression in multiple cancers using a novel npc2 monoclonal antibody. PloS one 2013;8:e77586.

133 Williams KC, McNeilly RE, Coppolino MG: Snap23, syntaxin4, and vesicle-associated membrane protein 7 (vamp7) mediate trafficking of membrane type 1-matrix metalloproteinase (mt1-mmp) during invadopodium formation and tumor cell invasion. Molecular biology of the cell 2014;25:2061-2070.

134 Sevilya Z, Leitner-Dagan Y, Pinchev M, Kremer R, Elinger D, Lejbkowicz F, Rennert HS, Freedman LS, Rennert G, Paz-Elizur T, Livneh Z: Development of ape1 enzymatic DNA repair assays: Low ape1 activity is associated with increase lung cancer risk. Carcinogenesis 2015;36:982-991.

135 Wei W, He XF, Qin JB, Su J, Li SX, Liu Y, Zhang Y, Wang W: Association between the ogg1 ser326cys and apex1 asp148glu polymorphisms and lung cancer risk: A meta-analysis. Mol Biol Rep 2012;39:11249-11262.

136 Hu D, Lin X, Zhang H, Zheng X, Niu W: Apex nuclease (multifunctional DNA repair enzyme) 1 gene asp148glu polymorphism and cancer risk: A meta-analysis involving 58 articles and 48903 participants. PloS one 2013;8:e83527.

137 Shukla VK, das PC, Dixit R, Bhartiya SK, Basu S, Raman MJ: Study of ap endonuclease (apex1/ref1), a DNA repair enzyme, in gallbladder carcinoma. Anticancer Res 2012;32:1489-1492.

138 Kim MH, Kim HB, Yoon SP, Lim SC, Cha MJ, Jeon YJ, Park SG, Chang IY, You HJ: Colon cancer progression is driven by apex1-mediated upregulation of jagged. J Clin Invest 2013;123:3211-3230.

139 Kasahara M, Osawa K, Yoshida K, Miyaishi A, Osawa Y, Inoue N, Tsutou A, Tabuchi Y, Tanaka K, Yamamoto M, Shimada E, Takahashi J: Association of mutyh gln324his and apex1 asp148glu with colorectal cancer and smoking in a japanese population. Journal of experimental & clinical cancer research : CR 2008;27:49.

140 Jing B, Wang J, Chang WL, Li B, Chen J, Niu YJ: Association of the polymorphism of ape1 gene with the risk of prostate cancer in chinese han population. Clinical laboratory 2013;59:163-168.

141 Kuasne H, Rodrigues IS, Losi-Guembarovski R, Reis MB, Fuganti PE, Gregorio EP, Libos Junior F, Matsuda HM, Rodrigues MA, Kishima MO, Colus IM: Base excision repair genes xrcc1 and apex1 and the risk for prostate cancer. Mol Biol Rep 2011;38:1585-1591.

142 Mahjabeen I, Baig RM, Sabir M, Kayani MA: Genetic and expressional variations of apex1 are associated with increased risk of head and neck cancer. Mutagenesis 2013;28:213-218.

143 Yang J, Yang D, Cogdell D, Du X, Li H, Pang Y, Sun Y, Hu L, Sun B, Trent J, Chen K, Zhang W: Apex1 gene amplification and its protein overexpression in osteosarcoma: Correlation with recurrence, metastasis, and survival. Technology in cancer research & treatment 2010;9:161-169.

144 L'Esperance S, Popa I, Bachvarova M, Plante M, Patten N, Wu L, Tetu B, Bachvarov D: Gene expression profiling of paired ovarian tumors obtained prior to and following adjuvant chemotherapy: Molecular signatures of chemoresistant tumors. International journal of oncology 2006;29:5-24.

145 Yu J, Liang QY, Wang J, Cheng Y, Wang S, Poon TC, Go MY, Tao Q, Chang Z, Sung JJ: Zinc-finger protein 331, a novel putative tumor suppressor, suppresses growth and invasiveness of gastric cancer. Oncogene 2013;32:307-317.

146 Mendez E, Lohavanichbutr P, Fan W, Houck JR, Rue TC, Doody DR, Futran ND, Upton MP, Yueh B, Zhao LP, Schwartz SM, Chen C: Can a metastatic gene expression profile outperform tumor size as a predictor of occult lymph node metastasis in oral cancer patients? Clinical cancer research : an official journal of the American Association for Cancer Research 2011;17:2466-2473.

147 Kim J, Hong SJ, Park JH, Park SY, Kim SW, Cho EY, Do IG, Joh JW, Kim DS: Expression of cystathionine beta-synthase is downregulated in hepatocellular carcinoma and associated with poor prognosis. Oncology reports 2009;21:1449-1454.

148 Cheng TY, Makar KW, Neuhouser ML, Miller JW, Song X, Brown EC, Beresford SA, Zheng Y, Poole EM, Galbraith RL, Duggan DJ, Habermann N, Bailey LB, Maneval DR, Caudill MA, Toriola AT, Green R, Ulrich CM: Folate-mediated one-carbon metabolism genes and interactions with nutritional factors on colorectal cancer risk: Women's health initiative observational study. Cancer 2015;121:3684-3691.

149 Tu XH, Huang SX, Li WS, Song JX: [correlation of methylation of cpg island in cystathionine beta synthase promoter and clinicopathological features in colorectal cancer]. Zhonghua zhong liu za zhi [Chinese journal of oncology] 2013;35:351-355.

150 Chakraborty PK, Xiong X, Mustafi SB, Saha S, Dhanasekaran D, Mandal NA, McMeekin S, Bhattacharya R, Mukherjee P: Role of cystathionine beta synthase in lipid metabolism in ovarian cancer. Oncotarget 2015;6:37367-37384.

151 Hellmich MR, Szabo C: Hydrogen sulfide and cancer. Handbook of experimental pharmacology 2015;230:233-241.

152 Hellmich MR, Coletta C, Chao C, Szabo C: The therapeutic potential of cystathionine beta-synthetase/hydrogen sulfide inhibition in cancer. Antioxidants & redox signaling 2015;22:424-448.

153 Szabo C, Coletta C, Chao C, Modis K, Szczesny B, Papapetropoulos A, Hellmich MR: Tumor-derived hydrogen sulfide, produced by cystathionine-beta-synthase, stimulates bioenergetics, cell proliferation, and angiogenesis in colon cancer. Proceedings of the National Academy of Sciences of the United States of America 2013;110:12474-12479.

154 Bhattacharyya S, Saha S, Giri K, Lanza IR, Nair KS, Jennings NB, Rodriguez-Aguayo C, Lopez-Berestein G, Basal E, Weaver AL, Visscher DW, Cliby W, Sood AK, Bhattacharya R, Mukherjee P: Cystathionine beta-synthase (cbs) contributes to advanced ovarian cancer progression and drug resistance. PloS one 2013;8:e79167.

155 Xia FD, Wang ZL, Chen HX, Huang Y, Li JD, Wang ZM, Li XY: Differential expression of iqgap1/2 in hepatocellular carcinoma and its relationship with clinical outcomes. Asian Pacific journal of cancer prevention : APJCP 2014;15:4951-4956.

156 White CD, Khurana H, Gnatenko DV, Li Z, Odze RD, Sacks DB, Schmidt VA: Iqgap1 and iqgap2 are reciprocally altered in hepatocellular carcinoma. BMC gastroenterology 2010;10:125.

157 Zeng H, Yu H, Lu L, Jain D, Kidd MS, Saif MW, Chanock SJ, Hartge P, PanScan C, Risch HA: Genetic effects and modifiers of radiotherapy and chemotherapy on survival in pancreatic cancer. Pancreas 2011;40:657-663.

158 Ghaleb AM, Bialkowska AB, Snider AJ, Gnatenko DV, Hannun YA, Yang VW, Schmidt VA: Iq motif-containing gtpase-activating protein 2 (iqgap2) is a novel regulator of colonic inflammation in mice. PloS one 2015;10:e0129314.

159 Jin SH, Akiyama Y, Fukamachi H, Yanagihara K, Akashi T, Yuasa Y: Iqgap2 inactivation through aberrant promoter methylation and promotion of invasion in gastric cancer cells. International journal of cancer Journal international du cancer 2008;122:1040-1046.

160 Zupancic K, Blejec A, Herman A, Veber M, Verbovsek U, Korsic M, Knezevic M, Rozman P, Turnsek TL, Gruden K, Motaln H: Identification of plasma biomarker candidates in glioblastoma using an antibody-array-based proteomic approach. Radiol Oncol 2014;48:257-266.

161 Liu Z, Zhang J, Wu L, Liu J, Zhang M: Overexpression of gnao1 correlates with poor prognosis in patients with gastric cancer and plays a role in gastric cancer cell proliferation and apoptosis. International journal of molecular medicine 2014;33:589-596.

162 Moore LE, Malats N, Rothman N, Real FX, Kogevinas M, Karami S, Garcia-Closas R, Silverman D, Chanock S, Welch R, Tardon A, Serra C, Carrato A, Dosemeci M, Garcia-Closas M: Polymorphisms in one-carbon metabolism and trans-sulfuration pathway genes and susceptibility to bladder cancer. International journal of cancer Journal international du cancer 2007;120:2452-2458.

163 Vanaja DK, Ehrich M, Van den Boom D, Cheville JC, Karnes RJ, Tindall DJ, Cantor CR, Young CY: Hypermethylation of genes for diagnosis and risk stratification of prostate cancer. Cancer investigation 2009;27:549-560.

164 Oeggerli M, Tian Y, Ruiz C, Wijker B, Sauter G, Obermann E, Guth U, Zlobec I, Sausbier M, Kunzelmann K, Bubendorf L: Role of kcnma1 in breast cancer. PloS one 2012;7:e41664.

165 Bloch M, Ousingsawat J, Simon R, Schraml P, Gasser TC, Mihatsch MJ, Kunzelmann K, Bubendorf L: Kcnma1 gene amplification promotes tumor cell proliferation in human prostate cancer. Oncogene 2007;26:2525-2534.

166 Khaitan D, Sankpal UT, Weksler B, Meister EA, Romero IA, Couraud PO, Ningaraj NS: Role of kcnma1 gene in breast cancer invasion and metastasis to brain. BMC cancer 2009;9:258.

167 Ibeawuchi C, Schmidt H, Voss R, Titze U, Abbas M, Neumann J, Eltze E, Hoogland AM, Jenster G, Brandt B, Semjonow A: Exploring prostate cancer genome reveals simultaneous losses of pten, fas and papss2 in patients with psa recurrence after radical prostatectomy. Int J Mol Sci 2015;16:3856-3869.

168 Wang X, Ju L, Fan J, Zhu Y, Liu X, Zhu K, Wu M, Li L: Histone h3k4 methyltransferase mll1 regulates protein glycosylation and tunicamycin-induced apoptosis through transcriptional regulation. Biochimica et biophysica acta 2014;1843:2592-2602.

169 Koie T, Yamamoto H, Imai A, Hatakeyama S, Yoneyama T, Hashimoto Y, Ohyama C: Significance of preoperative butyrylcholinesterase as an independent predictor of overall survival in patients with renal clear cell carcinoma treated with nephrectomy. J Clin Oncol 2014;32

170 Koie T, Ohyama C, Yamamoto H, Hatakeyama S, Imai A, Yoneyama T, Hashimoto Y, Kitayam M, Hirota K: Significance of preoperative butyrylcholinesterase as an independent predictor of survival in patients with muscle-invasive bladder cancer treated with radical cystectomy. Urologic oncology 2014;32:820-825.

171 Koie T, Ohyama C, Hatakeyama S, Imai A, Yoneyama T, Hashimoto Y, Yoneyama T, Tobisawa Y, Hosogoe S, Yamamoto H, Kitayama M, Hirota K: Significance of preoperative butyrylcholinesterase as an independent predictor of biochemical recurrence-free survival in patients with prostate cancer treated with radical prostatectomy. International journal of clinical oncology 2015

172 Castillo-Gonzalez AC, Nieto-Ceron S, Pelegrin-Hernandez JP, Montenegro MF, Noguera JA, Lopez-Moreno MF, Rodriguez-Lopez JN, Vidal CJ, Hellin-Meseguer D, Cabezas-Herrera J: Dysregulated cholinergic network as a novel biomarker of poor prognostic in patients with head and neck squamous cell carcinoma. BMC cancer 2015;15:385.

173 Prabhu K, Naik D, Ray S, Vadiraj, Rao A, Kamath A: Significance of serum butyrylcholinesterase levels in oral cancer. The Australasian medical journal 2011;4:374-378.

174 Chianeh YR, Manjunath R, Prabhu K, Fernandes D, Vidyasagar M, Kamath A: Protein thiols and butryrylcholinestrase in saliva of oral cancer patients. Indian journal of clinical biochemistry : IJCB 2014;29:238-241.

175 Racz A, Brass N, Heckel D, Pahl S, Remberger K, Meese E: Expression analysis of genes at 3q26-q27 involved in frequent amplification in squamous cell lung carcinoma. European journal of cancer 1999;35:641-646.

176 Luo Q, Wang C, Jin G, Gu D, Wang N, Song J, Jin H, Hu F, Zhang Y, Ge T, Huo X, Chu W, Shu H, Fang J, Yao M, Gu J, Cong W, Qin W: Lifr functions as a metastasis suppressor in hepatocellular carcinoma by negatively regulating phosphoinositide 3-kinase/akt pathway. Carcinogenesis 2015;36:1201-1212.

177 Guo H, Cheng Y, Martinka M, McElwee K: High lifr expression stimulates melanoma cell migration and is associated with unfavorable prognosis in melanoma. Oncotarget 2015;6:25484-25498.

178 Kim JC, Ha YJ, Roh SA, Choi EY, Yoon YS, Kim KP, Hong YS, Kim TW, Cho DH, Kim SY, Kim YS: Feasibility of proposed single-nucleotide polymorphisms as predictive markers for targeted regimens in metastatic colorectal cancer. Br J Cancer 2013;108:1862-1869.

179 Chen D, Sun Y, Wei Y, Zhang P, Rezaeian AH, Teruya-Feldstein J, Gupta S, Liang H, Lin HK, Hung MC, Ma L: Lifr is a breast cancer metastasis suppressor upstream of the hippo-yap pathway and a prognostic marker. Nat Med 2012;18:1511-1517.

180 Salm F, Dimitrova V, von Bueren AO, Cwiek P, Rehrauer H, Djonov V, Anderle P, Arcaro A: The phosphoinositide 3-kinase p110alpha isoform regulates leukemia inhibitory factor receptor expression via c-myc and mir-125b to promote cell proliferation in medulloblastoma. PloS one 2015;10:e0123958.

181 Yi Y, Nandana S, Case T, Nelson C, Radmilovic T, Matusik RJ, Tsuchiya KD: Candidate metastasis suppressor genes uncovered by array comparative genomic hybridization in a mouse allograft model of prostate cancer. Molecular cytogenetics 2009;2:18.

182 Lee YY, Li CF, Lin CY, Lee SW, Sheu MJ, Lin LC, Chen TJ, Wu TF, Hsing CH: Overexpression of cps1 is an independent negative prognosticator in rectal cancers receiving concurrent chemoradiotherapy. Tumour biology : the journal of the International Society for Oncodevelopmental Biology and Medicine 2014;35:11097-11105.

183 Abu-Zeid RM, Farid RM: Role of hepatocyte paraffin 1 antigen in the course of colorectal carcinogenesis. International journal of physiology, pathophysiology and pharmacology 2013;5:177-183.
